# The Relative Caloric Prices of Healthy and Unhealthy Foods Differ Systematically across Income Levels and Continents

**DOI:** 10.1093/jn/nxz158

**Published:** 2019-07-23

**Authors:** Derek D Headey, Harold H Alderman

**Affiliations:** International Food Policy Research Institute, Washington DC, USA

**Keywords:** food prices, undernutrition, obesity, food systems, dietary patterns

## Abstract

**Background:**

Relative prices of healthy/unhealthy foods have been implicated in the obesity epidemic, but never extensively quantified across countries or empirically linked to undernutrition.

**Objectives:**

This study compared relative caloric prices (RCPs) for different food categories across 176 countries and ascertained their associations with dietary indicators and nutrition outcomes.

**Methods:**

We converted prices for 657 standardized food products from the 2011 International Comparison Program into caloric prices using USDA Food Composition tables. We classified products into 21 specific food groups. We constructed RCPs as the ratio of the 3 cheapest products in each food group, relative to the weighted cost of a basket of starchy staples. We analyzed RCP differences across World Bank income levels and regions and used cross-country regressions to explore associations with Demographic Health Survey dietary indicators for women 15–49 y old and children 12–23 mo old and with WHO indicators of the under-5 stunting prevalence and adult overweight prevalence.

**Results:**

Most noncereal foods were relatively cheap in high-income countries, including sugar- and fat-rich foods. In lower-income countries, healthy foods were generally expensive, especially most animal-sourced foods and fortified infant cereals (FICs). Higher RCPs for a food predict lower consumption among children for 7 of 9 food groups. Higher milk and FIC prices were positively associated with international child stunting patterns: a 1-SD increase in milk prices was associated with a 2.8 percentage point increase in the stunting prevalence. Similarly, a 1-SD increase in soft drink prices was associated with a reduction in the overweight prevalence of ∼3.6 percentage points.

**Conclusions:**

Relative food prices vary systematically across countries and partially explain international differences in the prevalences of undernutrition and overweight adults. Future research should focus on how to alter relative prices to achieve better dietary and nutrition outcomes.

## Introduction

Child and maternal malnutrition and poor diets rank first and second, respectively, among level 2 risk factors for disability-adjusted life-years in the 2015 Global Burden of Disease Study, while a high BMI ranks sixth (1). In richer countries, the excess consumption of sugar, salt, meat, and *trans*-fatty acids is regarded as a major risk factor for noncommunicable diseases (2). Underconsumption of both macronutrients and micronutrients remains a risk factor for child mortality in the world's poorest countries (3). At the same time, many low- and middle-income countries are characterized by a double burden of malnutrition, with obesity rates rising sharply even as undernutrition persists (4, 5).

The causes of poor diets are complex. While nutritional knowledge and a range of behavioral and cultural factors clearly play a role (6), the affordability of healthy and unhealthy foods is an important determinant of dietary patterns. Policies that alter relative prices through trade, agricultural research, taxes, or subsidies are often regarded as cost-effective instruments for improving diets in both rich (7–10) and poor (11) countries alike.

Individuals in poorer countries have less dietary diversity than those in richer countries (12, 13). Self-evidently, low incomes constrain how much food poor households can buy. Yet for any given household's food budget, relative prices also influence which foods will be purchased. If, for example, dairy products are expensive relative to starchy staples whilst fish products are less so, diversification out of staples into fish is more likely than diversification into dairy products. Systematic food price dispersion across products and countries may play a major role in determining global dietary patterns and related nutrition outcomes.

The relative cheapness of calorie-dense processed foods has been implicated in the high rates of obesity observed in upper-income countries (14, 15), as well as the rapid increases in obesity observed in transition economies, such as China (16, 17). However, relatively little is known about the affordability of sugar, oils/fats, and calorie-dense processed foods in low- and middle-income countries or about the affordability of the nutrient-rich foods deemed essential for linear growth and cognitive development in early childhood, particularly animal-sourced foods (ASFs) (18). Previous research has analyzed price trends for specific foods in a single country, city, subnational region, or, occasionally, across a set of countries (15, 19–24). However, no studies have examined the structure of price differences globally and how these pricing structures might contribute to patterns in nutrition outcomes. In this study, we exploited a unique global data set of national prices for standardized food products to explore global patterns of relative prices and their relationship to dietary patterns, as well as child stunting and adult obesity.

## Methods

### Data

#### Description of the 2011 International Comparison Program price data

Our primary source of food price data was the 2011 International Comparison Program (ICP) survey (25). The ICP is a worldwide initiative under the auspices of the United Nations Statistical Commission and is the main statistical resource for comparing standards of living across the world, including gross domestic product (GDP) and poverty rates. A key mandate of the ICP is to survey prices of highly standardized (comparable) goods and services that are widely consumed across a region or globally. To do so, the World Bank and coordinating regional bodies asked national statistical agencies to collect consumer prices for an extensive list of standardized food and beverage products, some of which formed part of a global list of widely consumed products, whilst others are region-specific products. National statistical agencies were then tasked with utilizing nationally representative surveys of retail outlets to populate this list. Prices from different locations were then weighted to construct national averages representative of the country. Details of these steps and the challenges involved are provided in the 2011 ICP report (25). After excluding beverages and condiments with low or uncertain calorie contents, we utilized a list of 657 foods and beverages pertaining to 176 countries. **Supplemental Table 1** provides a list of all 657 food standard-definition products used in this analysis, and **Supplemental Table 2** provides meta-data on the survey frame used to collect price data in each country. In 2 instances, we supplemented ICP price data with consumer price data, since appropriate rice price data were missing for Japan and South Korea. Likewise, price data for wheat flour was imputed for the United States, Sweden, Norway, Macedonia, Japan, Guinea, and Bermuda using regional average prices in international dollars.

For the purposes of price comparisons, the most attractive feature of ICP price data is the high degree of standardization of food and beverage products: ICP definitions of food products refer to quantity, quality, weight, packing, processing, and other features of these products (e.g., “common brands”). This allows us to have a high degree of confidence that apples are being compared to apples and oranges to oranges.

#### Conceptualization and construction of relative food price indicators

We used ICP data to measure the ratio of the price of 1 calorie of a given food to the price of 1 calorie of a representative basket of starchy staple food in each country. These relative caloric prices (RCPs) have a simple interpretation: an RCP of 5 for eggs implies that it is 5 times as expensive to obtain a calorie from eggs as it is to obtain a calorie from starchy staples. This RCP measure has several attractive properties. Conceptually, this captures the relative cost of diversifying out of staple cereals into any specific nonstaple food group. This construction does not, however, imply that households base their choices only on calories, since clearly a number of factors, including taste, texture, tradition, and appearance, as well as nutritional knowledge, come into play as well (6). Nor is the RCP a metric designed to value the nutritional quality or importance of different foods. However, development economists focusing on low-income countries (26), as well as obesity researchers focusing on high-income countries (14, 15), have shown that calorie costs help explain food consumption patterns among poorer populations. Finally, the use of a ratio circumvents the serious challenges in using exchange rates to make international price comparisons (exchange rates are determined by comparative costs of tradable goods/services, but many foods are not tradable): the RCP is unit-free and does not require adjustments for cost-of-living differences or generic inflation.

The estimation of these RCPs involved 4 steps. First, the prices of all foods were converted into a price per calorie using the USDA's Food Composition Database on the calorie content of different foods and related data on the edible portions of different foods (27). In practice, edible portions may vary across countries, as may calorie density, but in the absence of country-specific data on these factors we were required to apply food-specific estimates common to all countries.

Second, we adapted FAO Guidelines (28) for measuring household and individual dietary diversity to classify specific foods in the ICP list into 21 food groups, to capture the scope for substitution between nutritionally similar products (**Table 1**). For part of our analysis, we grouped some of these categories together to match dietary data (e.g., pulses were combined with nuts). We further classified these 21 food groups into 4 broad groups based on very basic product characteristics (Table 1):

1. Starchy staples, consisting of 9 categories of cereals and roots/tubers that are important sources of calories but are generally low in micronutrients and high-quality protein. These make up the denominator in the RCP.
2. Vegetal foods, consisting of vitamin A–rich fruits and vegetables, green leafy vegetables rich in minerals and micronutrients, other fruits and other vegetables, legumes and nuts (rich in protein), and fortified infant cereals (FICs) dense in calories, fat, protein, and various micronutrients.
3. ASFs, rich in high-quality protein and bioavailable iron, as well as choline, vitamin B-12, and, in the case of dairy, insulin-like growth factor-1 and calcium. This broad group also includes processed and unprocessed red meats.
4. Sugar-rich, salt-rich, and fat-rich foods, including raw sugar, as well as oils/fats and various processed foods rich in sugar (soft drinks, sugar-rich snacks), fat (oils/fats), and salt (potato chips).

**TABLE 1:** The type and number of standardized food products in the International Comparison Program price database

Food group	*n* (products)	Examples
Starchy staples		
Wheat	41	Various flours, pastas, noodles, European/Asian breads
Rice	36	Coarse, polished, broken, aromatic, white/brown; rice noodles
Maize	18	Maize flour and grains, white and yellow; Maizena; tortillas
Potato	3	Brown, white, frozen; sweet potato
Millet	5	Flour, whole grain, couscous, bajra
Sorghum	2	Red/white grains
Cassava	2	Cassava/manioc/yuka
Yam	2	Taro, malanga, yautia, tannia, macab
Oats	1	Rolled oats
Vegetal foods		
Vitamin A–rich fruits and vegetables	24	Mango, apricots, guava, papaya, pumpkin
Dark green leafy vegetables	11	Spinach, cassava leaves, pumpkin leaves, bean leaves
Other vegetables	57	40 types, fresh/dried/canned, domestic, imported
Other fruit	45	31 types, fresh/dried, domestic/imported
Nuts	15	Almonds, peanuts, hazelnuts, walnuts, cashew
Pulses	26	14 types, including Asian varieties, imported/domestic, fresh/dry/tin
Fortified infant cereals	6	Wheat, maize, and rice-based cereals
Animal-sourced foods		
Milk (bovine)	16	Liquid/powdered milk, various fat contents, cow/buffalo
Other dairy	33	Yogurt, cheddar, haloumi, kashkaval, mozzarella, labneh, curd
Eggs	7	Chicken eggs (various sizes), duck eggs
White meat	24	Chicken, duck; live animal, various cuts; modern/traditional
Red meat, unprocessed	66	Beef, veal, pork, lamb, goat, mutton; live animal, various cuts
Red meat, processed	10	Hams, sausages, canned meats
Fish/seafood	81	50 distinct species, fresh, fillet, smoked, dried, canned
Sugar-rich, salt-rich, and fat-rich foods		
Fats/oils	29	14 types of oils, various butters/ghee, animal fats
Sugar	10	White, brown, loose/cubes, powdered, different sizes
Soft drinks	10	Cola, lemonade, international/domestic brands
Juice	18	Apple, orange, tomato, lime, pineapple, mango, mixed
Sugary snacks	55	Biscuits, bars, cakes, ice creams, pastries, jams, sweeteners
Potato chips	4	Potato chips
Total (all foods)	657	

In a third step, we constructed a weighted index of the median prices of various staple foods. Specifically, we used 2011 FAO *Food Balance Sheet* data (http://www.fao.org/faostat/en/) to measure the share of total starchy staple calories supplied by each of the 9 starchy staples listed in Table 1. These calorie shares were then used as weights in an index that measured the cost of purchasing 1000 calories of this basket of starchy staples. For example, India's starchy staple calories were derived principally from rice (49%) and wheat (35%), followed by coarse grains (12%) and potatoes (3%). The ICP data for India includes 26 types of rice products, 19 types of wheat-based products, 8 types of coarse grains, and 3 potato products, and the median price in each food group was used as the representative price of that food.

As the denominator in the RCPs, this starchy staple index has several desirable properties. First, it incorporates the fact that most countries have multiple staple foods, such as rice and wheat in India and China or cassava, maize, and rice in many African countries. Second, the use of the median price (rather than the minimum or average price) creates a more robust index that is less sensitive to outlying prices. Third, the use of median prices for each country factors in the higher cost of starchy staple calories in richer countries, where consumers tend to prefer more processed cereals (e.g., bread rather than wheat flour). **Supplemental Table 3** reports the value of this starchy staple index for World Bank income levels and major regions, measured as international dollars per 1000 kcal. This cost varies from a mean of $0.42 in low-income countries to $0.55 and $0.82 in lower middle– and upper middle–income countries, respectively, and $1.16 in high-income countries.

In the fourth and final step, for each of the 20 non-staple food groups, we estimated the average of the 3 cheapest specific food products in the group and divided that by the starchy staple caloric price index. As with the starchy staple approach, selecting multiple products in each food group ensured some degree of robustness and reflected the fact that consumers will typically consume different products within a food group. For all food groups, we measured a ratio of the average caloric cost of the cheapest products in that group relative to the caloric cost of a country-specific basket of starchy staples.

### Analysis

We analyzed global patterns of RCPs for different food groups using STATA v14 (StataCorp). We analyzed population-weighted mean RCPs by World Bank income groups and major regions, with data for India and China reported separately. These are presented in heat maps, as well as geographical maps. In both the heat maps and the geographical maps, the RCPs were binned into 4 categories: cheap (RCP <2), relatively cheap (RCP 2–4), moderately expensive (RCP 4–8), and very expensive (RCP 8–40). The choice of these bins is somewhat arbitrary, but facilitates the comparison of RCPs across food groups.

Next, we conducted a robust regression analysis using the *rreg* command (which downweighs any outlying values) to examine whether RCPs for different foods are associated with dietary indicators and nutrition outcomes. For diets, there is a dearth of internationally comparable data, so we used indicators from the Demographic Health Surveys that captured whether a food group had been consumed over a recent period (29). For children 12–23 mo old, we estimated the prevalence of a 24-h recall of consumption of 9 relatively healthy nonstaple foods, which map precisely into various food groups listed in Table 1. These indicators were available for over 50 low- and middle-income countries. For women 15–49 y old, we had indicators of 7-d recalls of 10 food groups, including oils/fats and sweets, that also map well into various food groups in Table 1. However, food consumption for adult women was only available for approximately 25 countries. For each food group, we estimated robust regressions against the log of the corresponding RCP for that food group, as well as the log GDP per capita. These regressions reflect very simple economic demand specifications in which income and prices play a dominant role.

We also used robust regressions to explore whether RCPs for fat- and sugar-rich foods were associated with the overweight prevalence (BMI >25) for adults 25 y and older and whether the prices of various healthy foods were associated with a reduced stunting prevalence [height-for-age *z* scores (HAZ) <−2] among children 0–5 y old. Both sets of nutrition outcomes were sourced from the WHO for 2011 (30). We estimated unadjusted regressions, as well as adjusted regressions that include the per capita gross national product, the urban population share, the labor force participation rate for women *≥*15 y old, the literacy rate for women 15+ y old, and—in the case of stunting regressions only—the share of the population using improved sanitation, obtained from the World Bank (31). These regression samples were substantially smaller than the larger sample used in the descriptive analysis since some countries were missing the supplementary data used in the multivariable regression analysis. We also tested the robustness of these core results to the use of severe stunting (HAZ <−3) and mild stunting (HAZ <−1); to employing the least squares regressor instead of the robust regressor; and to the inclusion of different sets of RCPs as explanatory variables.

## Results

### Food price variation in the global sample

**Table 2** reports summary statistics on RCPs for the full sample, as well as for other variables used in the analysis. The second column reports the number of countries, with data for each indicator and with most foods well represented. Mean RCPs show striking differences across food groups. The cheapest sources of calories are oil/fats and sugar, both of which are cheaper sources of calories than starchy staples (i.e., RCP <1), while legumes/nuts and sugar-rich snacks are also relatively cheap (RCP <2). Red meat is also surprisingly cheap, while most fruit, vegetables, and ASF categories are moderately expensive on average (RCPs of 4–8), with dark green leafy vegetables a very expensive exception (mean RCP = 20.1). However, for almost all foods, the SDs, minima, and maxima reveal large price dispersion across countries.

**TABLE 2:** Summary statistics for the variables used in this study^1^

Variable	*n* (countries)	Means ± SD (range)
RCPs		
Dark green leafy vegetables	168	20.1 ± 10.8 (2.9–71.5)
Vitamin A–rich fruit and vegetables	176	7.9 ± 6.3 (2.3–77.3)
Other vegetables	176	4.8 ± 2.1 (1.6–14.1)
Other fruit	176	4.1 ± 2.2 (0.9–12.6)
Nuts	168	2.7 ± 2.9 (0.7–22.5)
Pulses	174	2.5 ± 3.0 (0.4–32.2)
Pulses/nuts	176	1.6 ± 1.0 (0.4–10)
Infant cereal	169	4.9 ± 4.1 (0.7–27.2)
Milk	176	4.5 ± 3.7 (1–17.8)
Other dairy	176	4.9 ± 3.9 (1.1–25.4)
Any dairy (milk, other dairy)	176	3.8 ± 3.0 (1–17.4)
Eggs	170	5.9 ± 4.3 (1.4–23.2)
White meat	176	5.1 ± 3.4 (1.2–18.9)
Red meat, unprocessed	176	3.2 ± 1.3 (1.3–9.6)
Red meat, processed	145	10.7 ± 8.1 (1.3–64.5)
Any red meat	176	2.8 ± 1.2 (1.1–9.6)
Fish	176	6.4 ± 3.3 (1.2–23.4)
Fats/oils	176	0.7 ± 0.5 (0.2–5.1)
Sugar	174	0.8 ± 0.7 (0.1–6.1)
Soft drinks	175	5.5 ± 3.9 (1.5–24.1)
Juice	151	8.5 ± 6.8 (1.5–36)
Sugar-rich snacks	176	1.9 ± 0.9 (0.7–5.6)
Potato chips	172	3.1 ± 2.1 (0.8–15.3)
Child diets (children 6–23 mo), % consumed in past 24 h		
Vitamin A–rich fruit and vegetables	56	51 ± 17.1 (19–84.6)
Dark green leafy vegetables	56	23 ± 13.4 (3.6–47.8)
Other fruit/vegetables	56	30.6 ± 17.5 (4.3–72.5)
Legumes/nuts	56	26 ± 17.6 (4.3–75.3)
Infant cereal	49	13 ± 11.5 (1.2–49)
Any dairy	56	41.5 ± 25.2 (5.6–91.8)
Eggs	56	24.1 ± 17.2 (3.4–70.6)
Fish	55	26.6 ± 19.9 (1–81)
Meat	56	29 ± 20.4 (3.7–95.2)
Women's diets, % consumed in past 7 d		
Vitamin A–rich fruit and vegetables	24	39.5 ± 16.4 (18–77)
Dark green leafy vegetables	26	45.8 ± 18.0 (10.6–84.9)
Other fruit/vegetables	25	38.1 ± 18.7 (12.9–78.8)
Legumes/nuts	26	39.3 ± 21.2 (8.9–89.8)
Any dairy	25	30.6 ± 27.5 (3.6–90.7)
Eggs	26	27.4 ± 21.5 (2.9–82.1)
Fish	25	38.4 ± 27.4 (7.4–82.9)
Meat	25	37.5 ± 21.7 (9.1–82.1)
Oils/fats	24	55.6 ± 20.8 (20.6–94)
Sugar-rich snacks	25	30.5 ± 20.2 (4.4–86.1)
Nutrition outcomes (prevalence), %		
Overweight prevalence (BMI >25), adults 15–49 y	162	44.3 ± 15.7 (15.3–71.2)
Stunting prevalence (HAZ <−2), children 0–59 mo	108	24.5 ± 13.6 (1.8–57.5)
Control variables		
GDP per capita, 2011 international dollars	169	19,274 ± 21,444 (617–132,515)
Urban population share, %	173	58.5 ± 23.3 (8.9–100)
Female literacy (women 15+ y), %	136	80.9 ± 22.1 (15.1–99.9)
Female labor force participation (18–65 y), %	161	53.2 ± 15.5 (14.6–86.5)
Open defecation, % households with no toilet	160	10 ± 16.1 (0–74.8)

1 GDP, gross domestic product; HAZ, height-for-age *z* score; RCP, relative caloric price.

### Price variations across regions and income groups

#### Vegetal foods

**FIGURE 1:**
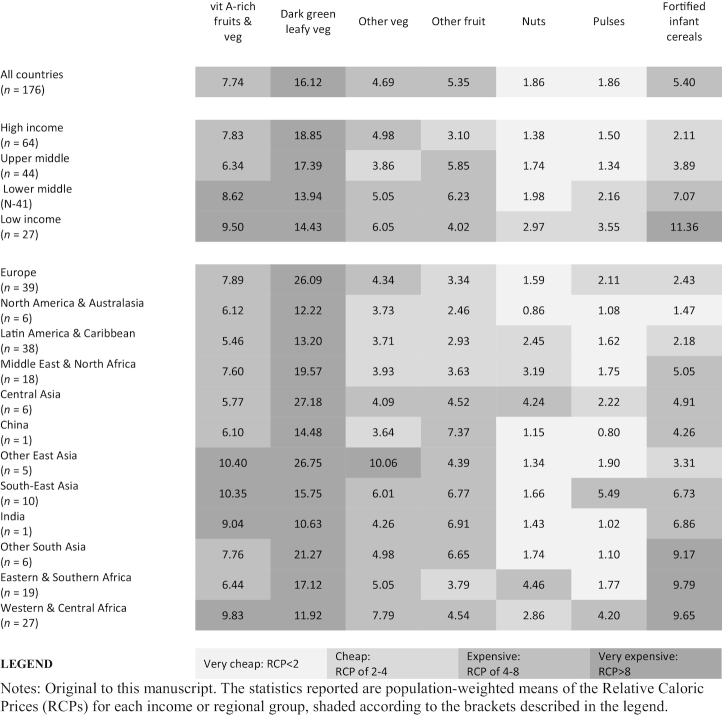
A heat map of RCPs of vegetal foods in 176 countries, by World Bank income levels and major regions, 2011 (population-weighted). The statistics reported are population-weighted means of the RCPs for each income or regional group, shaded according to the brackets described in the legend. RCP, relative caloric price; veg, vegetables; Vit, vitamin.

**FIGURE 2:**
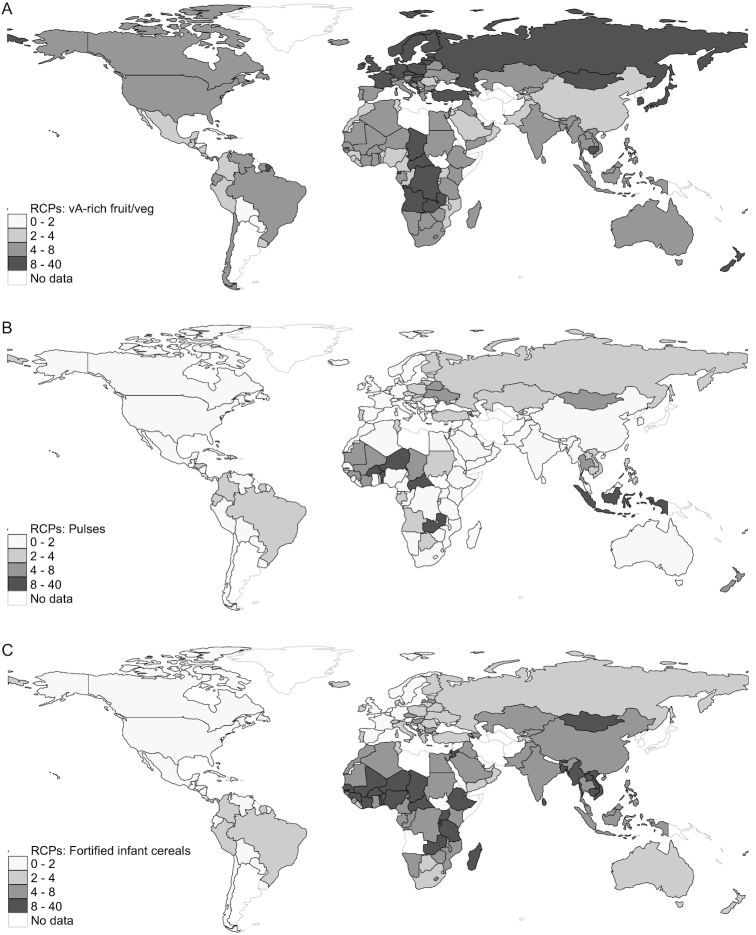
(A–C) Global variation in the RCPs of vitamin A–rich fruits and vegetables, pulses, and fortified infant cereals in 176 countries, 2011. The statistics reported are population-weighted means of the RCPs for each income or regional group, shaded according to the brackets described in the legend. RCP, relative caloric price; vA, vitamin A.

Plant-based foods varied substantially in their affordability across products, income levels, and regions (**Figures 1** and **2**). Vitamin A–rich fruits and vegetables were moderately expensive in most regions, were cheapest in Latin America and the Caribbean, and were most expensive in South-East Asia (Figure 2A). Dark green leafy vegetables were expensive in most regions, but relatively cheap in India and in Western and Central Africa, where spinach, as well as local products (e.g., cassava leaves), were relatively cheap. Other vegetables and fruits were moderately expensive in high-income countries and many middle-income regions, but were relatively expensive in much of Asia and sub-Saharan Africa. In contrast, nuts and pulses were typically classified into the very cheap and moderately cheap bins. Pulses were especially cheap in India and other South Asian countries, as well as in China and in Eastern and Southern Africa (Figure 2B). FICs, designed to supply complete nutrition to infants, were relatively cheap in high- and upper middle–income countries, but moderately expensive in lower middle–income countries and very expensive in low-income countries, where undernutrition in early childhood is most prevalent. In much of sub-Saharan Africa, for example, these products were almost 10 times as expensive per calorie as starchy staples, on average (Figure 2C).

#### Animal-sourced foods

**FIGURE 3:**
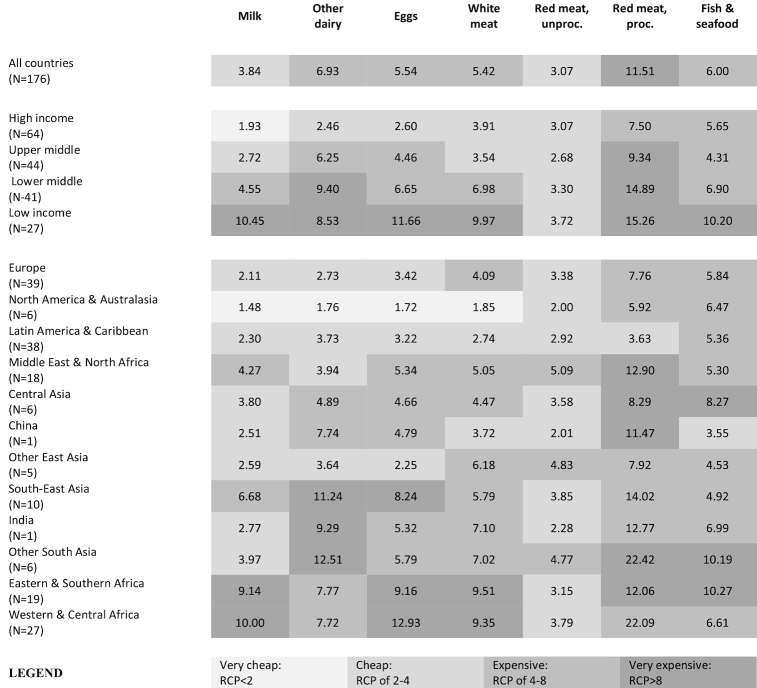
A heat map of RCPs of animal-sourced foods in 176 countries, grouped by World Bank income levels and major regions, 2011 (population-weighted means). The statistics reported are population-weighted means of the RCPs for each income or regional group, shaded according to the brackets described in the legend. proc., processed; RCP, relative caloric price; unproc., unprocessed.

**FIGURE 4:**
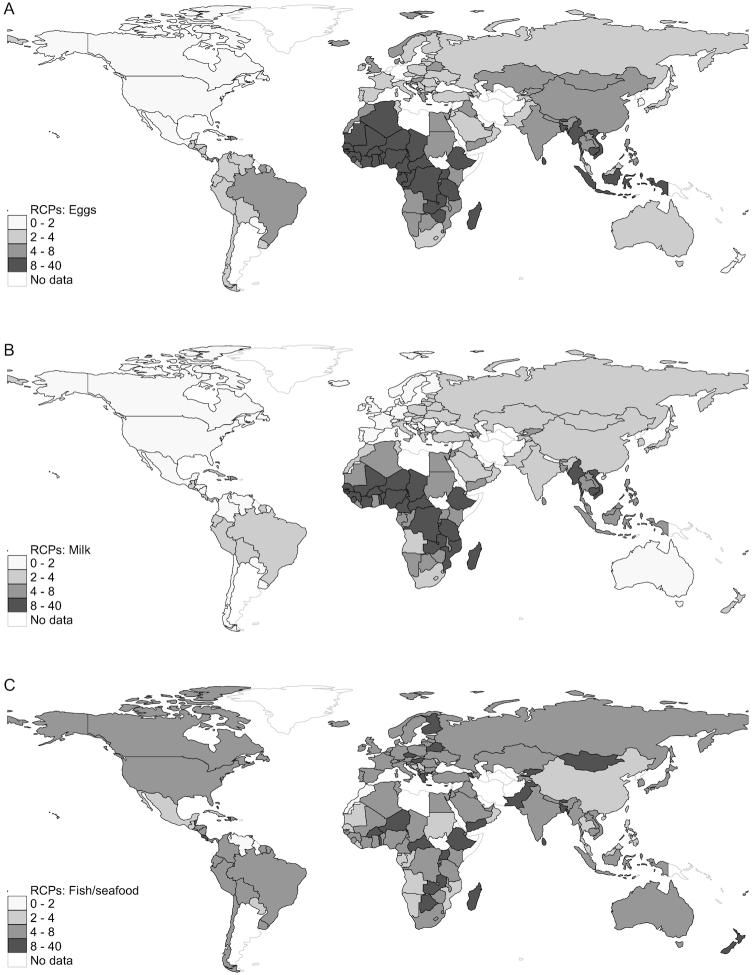
(A–C) Global variation in the RCPs of eggs, milk, and fish/seafood in 176 countries, 2011. RCP, relative caloric prices.

ASFs were typically more expensive in lower-income countries and poorer regions (**Figure 3**), although there are developing regions where certain ASFs are relatively cheap. The relative prices of dairy products, eggs, and white meat were strongly associated with income levels, being relatively cheap in high-income countries but very expensive in most low-income and lower middle–income countries and in sub-Saharan Africa (**Figure 4**A and B). India, however, was a notable outlier since dairy products were relatively cheap there (Figure 4B). Another contrast to the general pattern of higher ASF RCPs in poorer countries was unprocessed red meat, which was moderately cheap in all regions, partly reflecting its high calorie density (e.g., pork) and partly the affordability of lower-quality meat/organs in developing countries. Unprocessed red meat was especially cheap in China (RCP ∼2). Processed red meat was moderately expensive in high-income countries but very expensive elsewhere. Fish/seafood was moderately expensive in most regions but very expensive in low-income countries, on average. However, there was marked variation in fish/seafood RCPs across developing countries, and sometimes even within regions. For example, fish/seafood calories were 5–6 times more expensive than starchy staple calories in Latin America, the Middle East, and North Africa, but moderately cheap in East and South-East Asia (RCP <5). In Africa, fish/seafood were typically cheaper in coastal countries than in landlocked countries, and were classified in the moderately cheap category in countries such as Tanzania, Senegal, and Cameroon (Figure 4C).

#### Sugar-rich, salt-rich, and fat-rich foods

**FIGURE 5:**
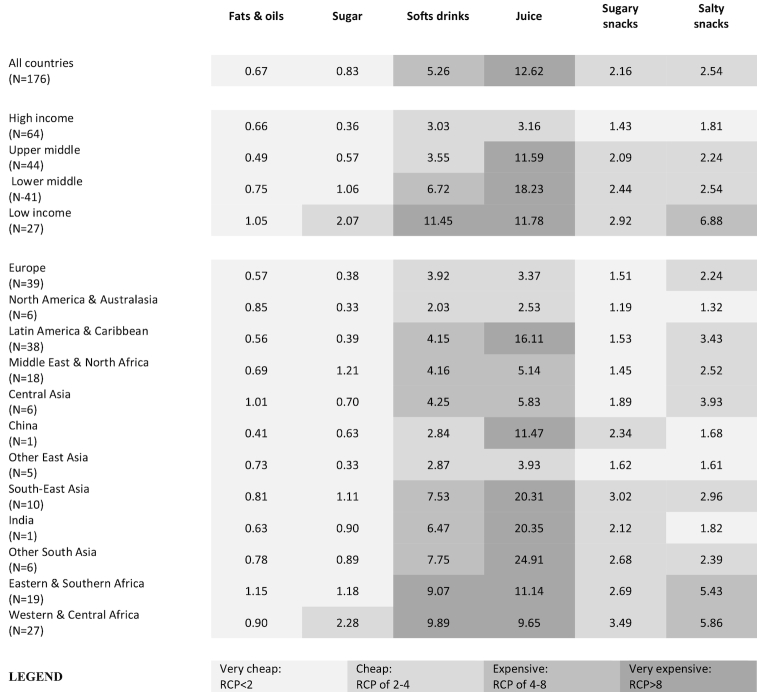
A heat map of RCPs of fat-rich, sugar-rich, and salt-rich foods in 176 countries, by World Bank income levels and major regions, 2011 (population-weighted means). The statistics reported are population-weighted means of the RCPs for each income or regional group, shaded according to the brackets described in the legend. RCP, relative caloric price.

**FIGURE 6:**
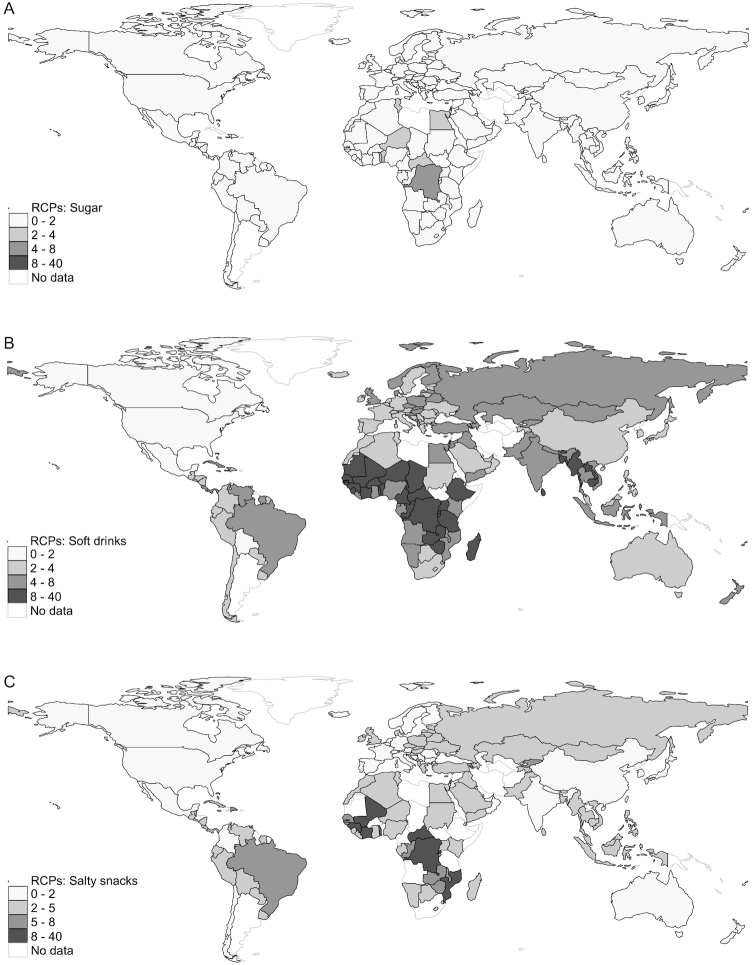
(A–C) Global variation in the RCPs of sugar, soft drinks, and salty snacks in 176 countries, 2011. The statistics reported are population-weighted means of the RCPs for each income or regional group, shaded according to the brackets described in the legend. RCP, relative caloric price.

Oils/fats were notably very cheap in all regions and were typically a cheaper source of calories than starchy staples (**Figure 5**). Sugar was also extremely cheap, although it was more expensive in lower-income settings and moderately expensive in several African countries (**Figure 6**A). Soft drinks were relatively cheap in high-income countries (but very cheap in North America and Australasia), moderately cheap in upper middle–income countries (and in Latin America, the Middle East, and North Africa), moderately expensive in lower middle–income countries and regions, and very expensive in low-income countries (mostly in sub-Saharan Africa; Figure 6B). Juice followed similar patterns, though it tended to be somewhat more expensive than soft drinks, on average. In contrast, sugary snacks were moderately cheap in high-income countries, but tended to be moderately or very expensive in other income brackets and in most developing regions. However, potato chips were a very cheap source of calories in high-income countries and moderately cheap in most upper middle– and lower middle–income countries and regions, but were very cheap in China, India, and other East Asian countries (Figure 6C).

#### Associations between RCPs and dietary patterns for adult women and young children

**Table 3** reports robust regression results for associations between the consumption of various food groups in the past 24 h among children 12–23 mo old and the log of the corresponding RCP, as well as the log of GDP per capita. Table 3 also reports the mean consumption prevalence for these foods, as well as mean RCPs in this sample of low- and middle-income Demographic Health Survey countries. Despite the relatively small sample, RCPs were significant predictors of consumption patterns among young children: the coefficient on each food group's respective RCP (own price) was negative and statistically significant at the 5% level for 7 of 9 food groups. The only exceptions were vitamin A–rich fruits and vegetables and pulses and nuts. However, in these 2 instances we noted that the coefficients on the log GDP per capita were also statistically nonsignificant, suggesting that economic factors are not the principal determinants of consumption of these foods. For the other food groups, consumption patterns were highly responsive to prices. For example, for the adjusted model, a 1-SD increase in the price of eggs predicted a 9.2 point reduction in the percentage of children consuming eggs in the past 24 h.

**TABLE 3:** Cross-country robust regressions^1^

	Vitamin A–rich fruit/vegetables	Dark green leafy vegetables	Other fruit/vegetables	Pulses and nuts	Infant cereals	Dairy	Eggs	Fish	Meat
Own price (RCP), log	−0.04	−0.08**	−0.13**	0.03	−0.08***	−0.09*	−0.12***	−0.15***	−0.10^#^
	(−0.15, 0.06)	(−0.14, −0.03)	(−0.21, −0.05)	(−0.08, 0.15)	(−0.12, −0.04)	(−0.17, −0.01)	(−0.17, −0.06)	(−0.24, −0.07)	(−0.21, 0.00)
GDP per capita, log	−0.03	−0.04	0.11***	0.01	0.04**	0.21***	0.09***	−0.08**	0.13***
	(−0.09, 0.03)	(−0.08, 0.00)	(0.06, 0.15)	(−0.05, 0.06)	(0.01, 0.07)	(0.16, 0.26)	(0.06, 0.13)	(−0.13, −0.03)	(0.09, 0.18)
Mean consumption, %	51.0	23.0	30.6	26.0	13.0	41.5	24.1	26.6	29.0
Mean RCP	7.9	14.6	3.1	1.6	7.7	7.2	8.6	7.1	3.2
*R^2^*	0.03	0.25	0.46	0.01	0.51	0.67	0.61	0.28	0.45
*n* (countries)	56	52	56	56	48	56	56	55	56

1 Regressions are of 24-h recall estimates of consumption prevalence of 9 different food groups among children 12–23 mo old against corresponding RCPs and GDP per capita (both in logs). All values are βs (95% CIs) derived via the robust regression command in STATA 14 ( *rreg*), which downweighs outlying observations, but results are qualitatively similar with the least squares estimator. ^#^*P* < 0.10; *P   < 0.05; ^**^P   < 0.01; ^***^P   < 0.001. GDP, gross domestic product; RCP, relative caloric price.

**Supplemental Table 4** reports analogous results for consumption prevalence estimates for women 15–49 y old. Here, the small sample contributed to imprecision, so fewer own price coefficients were statistically significant. However, the pattern of coefficient signs and magnitudes was generally similar (indeed, Pearson correlations between children's and women's consumption estimates are typically greater than 0.50) and the own price elasticities on ASFs were often particularly large in magnitude. A difference for women is that we had estimates of the consumption prevalence for oils/fats and sugar-rich sweets. Interestingly, for these 2 groups, the own price coefficients were statistically significant at the 5% level; were negative, as expected; and were large in magnitude.

#### Associations between RCPs and adult overweight prevalence

**Table 4** reports associations between RCPs for sugar, soft drinks, oils/fats, and salty snacks and the adult overweight prevalences, using unadjusted and adjusted models. The unadjusted models revealed statistically significant coefficients for all 4 unhealthy foods, although the associations are stronger for sugar and soft drink prices. The adjusted models suggest that the association between sugar/soft drink prices and excess body weight was robust (with both coefficients still significant at the 0.1% level), although the coefficients declined in magnitude by more than half relative to the unadjusted model. In contrast, the log RCP coefficients for oils/fats and salty snacks were no longer significant in the adjusted models. The adjusted model results for sugar and soft drink RCPs were also robust to controlling for oil/fats and salty snack RCPs (**Supplemental Table 5**) and to least squares regressions (**Supplemental Table 6**). Furthermore, the RCPs for sugar and sugar-rich foods were highly correlated with each other. The RCP coefficient for soft drinks from the adjusted model was also particularly large in magnitude; a 1-SD decrease in soft drink prices predicted a 3.9 point increase in the overweight prevalence.

**TABLE 4:** Cross-country robust regression estimates^1^

	Sugar RCP, logged	Soft drink RCP, logged	Oil/fat RCP, logged	Potato chip RCP, logged
Unadjusted model	−14.65*	−18.97*	−12.56*	−11.95*
	(−18.08, −11.21)	(−23.22, −14.73)	(−18.54, −6.57)	(−17.24, −6.65)
*R^2^*	0.40	0.42	0.14	0.16
*n* (countries)	115	115	115	115
Adjusted model	−4.57*	−6.10*	−1.33	0.67
	(−6.61, −2.54)	(−8.85, −3.35)	(−4.30, 1.64)	(−2.51, 3.85)
*R^2^*	0.85	0.86	0.79	0.79
*n* (countries)	115	115	115	115

1 Estimates are of the associations between the overweight prevalence among adults 25 y and older and the RCPs of various unhealthy foods in unadjusted and adjusted models. All values are βs (95% CIs) derived via the robust regression command in STATA 14 ( *rreg*), which downweighs outlying observations, although results are qualitatively similar with the least squares estimator. Unadjusted models specify no controls, while the adjusted model controls for GDP per capita, the urban population share, the labor force participation rate for women 15+ y old, and the literacy rate in women 15+ y old (all of which are specified in logs). *P   < 0.001. GDP, gross domestic product; RCP, relative caloric price.

### Associations between stunting prevalence and the prices of various healthy foods

**Table 5** reports tests of associations between child stunting and the relative prices of fresh cow's milk, chicken eggs, meat/fish, and FICs, since previous research has linked stunting reduction to children's intake of ASFs, particularly dairy (32, 33) and eggs (34). The unadjusted models revealed significant positive associations between the prices of these nutrient-rich foods and child stunting. However, the addition of the control variables reduced the magnitude of the coefficients quite markedly. In the adjusted models, the price of fresh cow's milk had a highly significant coefficient that was still relatively large in magnitude: a 1 standard deviation increase in milk prices predicted a 2.7 percentage point increase in stunting prevalence. Egg and FIC RCP coefficients were still significant at the 5% level in the adjusted model. **Supplemental Table 7** specified models with multiple RCPs in the same model. Strikingly, irrespective of the model, it was only the coefficient on milk prices that remained robustly statistically significant. **Supplemental Table 8** also compared results for moderate stunting (HAZ <−2) to severe (HAZ <−3) and mild stunting (HAZ <−3). Across all 3 measures, only the coefficients on the log of the RCP for dairy were robustly significant. Finally, **Supplemental Table 9** showed similar results for least squares regressions instead of robust regressions. In the adjusted least squares model, the dairy RCP coefficient was significant at the 5% level.

**TABLE 5:** Cross-country robust regression estimates^1^

	Milk RCP, logged	Egg RCP, logged	Meat/fish RCP, logged	Infant cereal RCP, logged
Unadjusted model	12.53***	11.60***	9.70**	11.36***
	(9.68, 15.38)	(8.21, 14.98)	(3.64, 15.75)	(8.33, 14.39)
*R^2^*	0.44	0.33	0.10	0.38
*n* (countries)	101	101	101	95
Adjusted model	4.79***	3.34*	0.48	3.14*
	(2.02, 7.56)	(0.42, 6.26)	(−3.52, 4.49)	(0.28, 5.99)
*R^2^*	0.75	0.72	0.70	0.72
*n* (countries)	101	101	101	94

1 Estimates are of the associations between stunting prevalence among children 0–5 y old and the RCPs of animal-sourced foods and fortified infant cereals in unadjusted and adjusted models. All values are βs (95% CIs) derived via the robust regression command in STATA 14 ( *rreg*), which downweighs outlying observations, although results are qualitatively similar with the least squares estimator. Unadjusted models specify no controls, while the adjusted model controls for GDP per capita, the urban population share, the labor force participation rate in women 15+ y, the literacy rate in 15+ y, and the share of the population not using toilets (open defecation) (all of which are specified in logs). *P   < 0.05; ^**^P   < 0.01; ^***^P   < 0.001. GDP, gross domestic product; RCP, relative caloric price.

In addition to the food groups used in Table 5, we also investigated whether RCPs for vitamin A–rich fruits and vegetables, dark green leafy vegetables, other fruit, and pulses/nuts were significantly associated with stunting rates (**Supplemental Table 10**). However, none of the RCPs for these foods yielded statistically significant positive coefficients in the adjusted models, and dark green leafy vegetable RCPs were negatively associated with the stunting prevalence.

## Discussion

In this paper, we describe international patterns in the relative affordability of various healthy and unhealthy foods (calories) via RCPs and explore whether RCPs for various foods explain the consumption of those foods by young children and women and, thus, child stunting and adult overweight prevalences, respectively.

We found that the RCPs of most healthy foods are substantially more expensive in poorer countries. Partial exceptions include milk in countries that are significant dairy producers (e.g., India), vitamin A–rich fruits and vegetables (which are sometimes cheaper in tropical countries), dark green leafy vegetables (which are cheaper in India and parts of sub-Saharan Africa than in high-income countries), and fish (cheaper in much of Asia and much of Central and Western Africa). These exceptions aside, most nutritious foods are expensive in lower-income countries. Eggs and fresh milk, for example, are often 10 times as expensive as starchy staples in caloric terms. Unsurprisingly, we found that higher ASF prices typically lead to less frequent consumption among young children.

Given that the demand for ASFs is constrained by low incomes in these countries, the most plausible explanation for high ASF prices is supply constraints. Many ASFs are highly perishable, especially fresh cow's milk and eggs; low-cost imports offer limited scope to bring prices down. Poor productivity in the dairy and poultry sectors of low-income countries, therefore, directly translates into high prices. Dairy production has some specific constraints. Dairy production is poorly suited to tropical climates and, in Africa, cattle ownership is severely constrained by tsetse flies (35). For countries with low potential for dairy production, the most viable means of increasing consumption will be to use dairy powder imports judiciously—including industrial reconstitution—in conjunction with efforts to stimulate domestic production and domestic demand for milk, a path which several Asian countries have followed (36). Industrial reconstitution can improve food safety (especially where reliable water at the household level is not assured), and also provides scope for fortification.

The high price of eggs is paradoxical, given that poultry are the most widely owned livestock in developing countries (37). However, homestead poultry production is hindered by diseases (such as Newcastle's) and low inputs. Countries that have achieved larger-scale commercial production with the use of improved feed, housing, and vaccinations, such as India, have seen marked declines in the prices of eggs and poultry products, even in the face of rising demand (38). Another striking result among ASFs is the relatively affordable price of fish in sub-Saharan Africa and Asia. Fish accounts for 22% of the protein intake in sub-Saharan Africa (39), where it is an important part of the diets of young children (40). Despite this, relatively little research has evaluated the nutritional impacts of aquaculture interventions in this region.

Among specialized infant foods, fortified cereals have attractive properties for children living in poor nutritional environments, including high macro- and micronutrient contents, high degrees of palatability for children only previously fed breastmilk, and minimal preparation times, although, as with reconstituted milk, the quality of the water supply needs to be considered. Despite these potential benefits, our results suggest that, in low-income regions, these products are 7 times more expensive than they are in high-income countries and are often 20–30 times as expensive as the unfortified staple cereals that are more commonly fed to infants in poor countries. Previous research has suggested that markets for FICs are uncompetitive because consumers have little trust in the nutrient content or safety of domestically produced infant cereals, leaving markets monopolized by more trusted international brands (41).

The second objective of this study was to examine whether food price dispersion across countries explains international patterns in excess body weight among adults and in inadequate linear growth among young children. We found that relative dairy and egg prices are strongly associated with international variation in stunting rates, consistent with an extensive literature linking dairy consumption to linear growth in young children (32, 33) and a recent control trial linking egg consumption to child growth in Ecuador (34). We also found that prices of FICs are strongly associated with reduced rates of stunting, consistent with survey-based studies linking the consumption of these products to the linear growth of young children (42). However, among these different results, dairy has the most robust associations with stunting.

We also found associations between the prices of various unhealthy foods and the overweight prevalence among adult populations, consistent with previous research from high-income countries suggesting that the relative cheapness of unhealthy calories is an important explanation of the obesity epidemic in higher-income countries (9, 10, 14, 15, 22). Our results on the tight link between sugar and sugar-rich food prices and the overweight prevalence among adults are consistent with a growing literature linking the consumption of these products to weight gain (43, 44).

Our study has several limitations. The price data are national averages that are intended to be representative although, in practice, most price surveys underrepresent smaller and more remote rural markets. Moreover, there are likely to be systematic RCP differences for rural and urban consumers and, indeed, for different socioeconomic strata. Rural consumers are also often producers of food and may consume their own produce, making market prices only indirectly relevant to their consumption decisions. However, high market prices may also encourage rural households to sell high-value foods for income generation, so that high prices still result in low consumption. We are also limited by only having data for 1 round of the ICP (2011), since earlier ICP rounds did not release the price data of individual goods. Our data, therefore, do not speak to price trends over time or to price variations within countries, both of which are important areas for future research. Nor can we address seasonal price fluctuations; in many places, fruits and vegetables are inexpensive in 1 season and virtually unavailable in another, and future research should address seasonal variations in prices, and particularly in poorer countries, where transport and storage systems are less developed.

Finally, our analysis of relationships between the national averages of prices and nutrition outcomes is a purely ecological analysis, which has well-known limitations for any causal inference. The current study is, to our knowledge, the first to document large and systematic differences in the relative prices of different healthy and unhealthy foods. In keeping with studies that document the importance of food prices for shaping food demand (45, 46), our study found suggestive evidence that differences in food prices may partially explain prevailing international patterns in child stunting and adult overweight/obesity. These findings raise important areas for future research on what explains food price variations across countries (and within countries) and on how to cost-effectively and appropriately alter relative prices through agricultural interventions (47), trade policies, or taxation (7–10).

In summary, this study demonstrates that the affordability of both healthy and unhealthy foods varies markedly across regions and levels of development, and that these variations in relative prices are strongly associated with nutrition outcomes. These findings raise an important agenda for future research: how best to alter these prices so as to shape better diets in rich and poor countries alike.

## Supplementary Material

nxz158_Supplemental_File

## Notes

This research was conducted under the “Advancing Research on Nutrition and Agriculture (AReNA)” project, Phase 1, supported by The Bill & Melinda Gates Foundation (investment ID OPP1112307) and the Consultative Group on International Agricultural Research (CGIAR) Program on Agriculture for Nutrition and Health.

Author disclosures: DDH and HHA, no conflicts of interest.

Supplemental Tables 1–10 are available from the “Supplementary data” link in the online posting of the article and from the same link in the online table of contents at https://academic.oup.com/jn/.

Abbreviations used: ASF, animal-sourced food; FIC, fortified infant cereal; GDP, gross domestic product; HAZ, height-for-age *z* score; ICP, International Comparison Program; RCP, relative caloric price.

## References

[1] Forouzanfar MH, Alexander L, Anderson HR, Bachman VF, Biryukov S, Brauer M, Burnett R, Casey D, Coates MM, Cohen A et al. Global, regional, and national comparative risk assessment of 79 behavioural, environmental and occupational, and metabolic risks or clusters of risks in 188 countries, 1990–2013: a systematic analysis for the Global Burden of Disease Study 2013. Lancet North Am Ed. 2015;386:2287–323. 10.1016/S0140-6736(15)00128-2 PMC4685753 26364544

[2] Wang YC, McPherson K, Marsh T, Gortmaker SL, Brown M Health and economic burden of the projected obesity trends in the USA and the UK. Lancet North Am Ed. 2011;378:815–25. 10.1016/S0140-6736(11)60814-3 21872750

[3] Black RE, Allen LH, Bhutta ZA, Caulfield LE, de Onis M, Ezzati M, Mathers C, Rivera J Maternal and child undernutrition: global and regional exposures and health consequences. Lancet. 2008;371:243–60. 18207566 10.1016/S0140-6736(07)61690-0

[4] Imamura F, Micha R, Khatibzadeh S, Fahimi S, Shi P, Powles J, Mozaffarian D Dietary quality among men and women in 187 countries in 1990 and 2010: a systematic assessment. Lancet Glob Health. 2015;3:e132–42. 25701991 10.1016/S2214-109X(14)70381-X PMC4342410

[5] Lim SS, Vos T, Flaxman AD, Danaei G, Shibuya K, Adair-Rohani H, AlMazroa MA, Amann M, Anderson HR, Andrews KG et al. A comparative risk assessment of burden of disease and injury attributable to 67 risk factors and risk factor clusters in 21 regions, 1990–2013: a systematic analysis for the Global Burden of Disease Study 2010. Lancet North Am Ed. 2013;380:2224–60. 10.1016/S0140-6736(12)61766-8 PMC4156511 23245609

[6] Dubois P, Griffith R, Nevo A Do prices and attributes explain international differences in food purchases? Am Econ Rev. 2014;104:832–67.

[7] Frieden TR, Dietz W, Collins J Reducing childhood obesity through policy change: acting now to prevent obesity. Health Aff. 2010;29:357–63. 10.1377/hlthaff.2010.0039 20194973

[8] Wang YC, Coxson P, Shen Y-M, Goldman L, Bibbins-Domingo K A penny-per-ounce tax on sugar-sweetened beverages would cut health and cost burdens of diabetes. Health Aff (Millwood). 2012;31:199–207. 22232111 10.1377/hlthaff.2011.0410

[9] Darmon N, Drewnowski A. Contribution of food prices and diet cost to socioeconomic disparities in diet quality and health: a systematic review and analysis. Nutr Rev. 2015;73:643–60. 26307238 10.1093/nutrit/nuv027 PMC4586446

[10] Maillot M, Darmon N, Darmon M, Lafay L, Drewnowski A Nutrient-dense food groups have high energy costs: an econometric approach to nutrient profiling. J Nutr. 2007;137:1815–20. 17585036 10.1093/jn/137.7.1815

[11] Herforth A, Ahmed S. The food environment, its effects on dietary consumption, and potential for measurement within agriculture-nutrition interventions. Food Security. 2015;7:505–20.

[12] Micha R, Khatibzadeh S, Shi P, Andrews KG, Engell RE, Mozaffarian D Global, regional and national consumption of major food groups in 1990 and 2010: a systematic analysis including 266 country-specific nutrition surveys worldwide. BMJ Open. 2015;5:e008705. 10.1136/bmjopen-2015-008705 PMC4593162 26408285

[13] Clements KW, Si J. Engel's law, diet diversity, and the quality of food consumption. Am J Agr Econ. 2018;100(1):1–22.

[14] Drewnowski A, Specter S Poverty and obesity: the role of energy density and energy costs. Am J Clin Nutr. 2004;79:6–16. 14684391 10.1093/ajcn/79.1.6

[15] Drewnowski A. The cost of US foods as related to their nutritive value. Am J Clin Nutr. 2010;92:1181–8. 20720258 10.3945/ajcn.2010.29300 PMC2954450

[16] Popkin BM. The nutrition transition and obesity in the developing world. J Nutr. 2001;131:871S–3S. 11238777 10.1093/jn/131.3.871S

[17] Lu Y, Goldman D. The effects of relative food prices on obesity – evidence from China: 1991–2006. National Bureau of Economic Research Working Paper Series, No. 15720 Cambridge, MA: National Bureau of Economic Research; 2010.

[18] Murphy S, Allen L. Nutritional importance of animal source foods. J Nutr. 2003;133:3932s–5s. 14672292 10.1093/jn/133.11.3932S

[19] Drewnowski A. The Nutrient Rich Foods Index helps to identify healthy, affordable foods. Am J Clin Nutr. 2010;91:1095S–101S. 20181811 10.3945/ajcn.2010.28450D

[20] Glanz K, Basil M, Maibach E, Goldberg J, Snyder DAN Why Americans eat what they do: taste, nutrition, cost, convenience, and weight control concerns as influences on food consumption. J Am Diet Assoc. 1998;98:1118–26. 9787717 10.1016/S0002-8223(98)00260-0

[21] Beydoun MA, Powell L, Wang Y The association of fast food, fruit and vegetable prices with dietary intakes among US adults: Is there modification by family income?. Social Science & Medicine. 2008;66:2218–29. 18313824 10.1016/j.socscimed.2008.01.018 PMC4863648

[22] Monsivais P, McLain J, Drewnowski A The rising disparity in the price of healthful foods: 2004–2008. Food Policy. 2010;35:514–20. 25411518 10.1016/j.foodpol.2010.06.004 PMC4234177

[23] Han E, Powell LM. Effect of food prices on the prevalence of obesity among young adults. Public Health. 2011;125:129–35. 21272902 10.1016/j.puhe.2010.11.014

[24] Grossman M, Tekin E, Wada R Food prices and body fatness among youths. National Bureau of Economic Research Working Paper Series, No. 19143 Cambridge, MA: National Bureau of Economic Research; 2013. 10.1016/j.ehb.2013.10.003 24246131

[25] World Bank. Purchasing power parities and the real size of world economies: a comprehensive report of the 2011 International Comparison Program. Washington, DC: World Bank; 2015.

[26] Subramanian S, Deaton A. The demand for food and calories. J Polit Econ. 1996;104:133–62.

[27] United States Department of Agriculture USDA food composition database. Washington, DC: United States Department of Agriculture; 2017.

[28] Kennedy G, Ballard T, Dop MC Guidelines for measuring household and individual dietary diversity. Rome, Italy: Food and Agriculture Organization; 2010.

[29] ICF International. The demographic and health surveys program. Calverton, MD: ICF International; 2017.

[30] Abarca-Gómez L, Abdeen ZA, Hamid ZA, Abu-Rmeileh NM, Acosta-Cazares B, Acuin C, Adams RJ, Aekplakorn W, Afsana K, Aguilar-Salinas CA et al. Worldwide trends in body-mass index, underweight, overweight, and obesity from 1975 to 2016: a pooled analysis of 2416 population-based measurement studies in 128.9 million children, adolescents, and adults. Lancet North Am Ed. 2017;390:2627−42. 10.1016/S0140-6736(17)32129-3 PMC5735219 29029897

[31] World Bank. World development indicators online. Washington, DC: The World Bank; 2017.

[32] de Beer H Dairy products and physical stature: a systematic review and meta-analysis of controlled trials. Econ Hum Biol. 2012;10:299–309. 21890437 10.1016/j.ehb.2011.08.003

[33] Iannotti L, Muehlhoff E, McMahon D Review of milk and dairy programmes affecting nutrition. J Dev Effect. 2013;5:82–115.

[34] Iannotti LL, Lutter CK, Stewart CP, Gallegos Riofrío CA, Malo C, Reinhart G, Palacios A, Karp C, Chapnick M, Cox K et al. Eggs in early complementary feeding and child growth: a randomized controlled trial. Pediatrics. 2017;140:e20163459. 28588101 10.1542/peds.2016-3459

[35] Alsan M. The effect of the TseTse fly on African development. Am Econ Rev. 2015;105:382–410.

[36] Food and Agriculture Organisation. Improved market access and smallholder dairy farmer participation for sustainable dairy development: Asia smallholder dairy development strategy and outline investment plan. Bangkok, Thailand: Food and Agriculture Organisation; 2008.

[37] Headey D, Hirvonen K. Is exposure to poultry harmful to child nutrition? An observational analysis for rural Ethiopia. PLOS One. 2016;11:e0160590. 27529178 10.1371/journal.pone.0160590 PMC4986937

[38] Narrod C, Tiongco M, Costales A Global poultry sector trends and external drivers of structural change. In: Theime O, Pilling D Poultry in the 21st Century: avian influenza and beyond. Rome, Italy: Food and Agriculture Organisation; 2007, p.21−48.

[39] Kaliba AR, Ngugi CC, Mackambo JM, Osewe KO, Senkondo E, Mnembuka BV, Amisah S Potential effect of aquaculture promotion on poverty reduction in Sub-Saharan Africa. Aquacult Int. 2007;15:445–59.

[40] Headey D, Hoddinott J, Hirvonen K Animal sourced foods and child stunting. Am J Agric Econ. 2018;100:1302−19. 10.1093/ajae/aay053 PMC7734193 33343003

[41] Masters WA, Nene MD, Bell W Nutrient composition of premixed and packaged complementary foods for sale in low- and middle-income countries: lack of standards threatens infant growth. Matern Child Nutr. 2017;13:e12421. 10.1111/mcn.12421 PMC6865969 28008727

[42] Diana A, Mallard S, Haszard J, Monik Purnamasari D, Nurulazmi I, Herliani PD, Irawan G, Gibson R, Houghton L Consumption of fortified infant foods reduces dietary diversity but has a positive effect on subsequent growth in infants from Sumedang district, Indonesia. PLOS One. 2017;12:e0175952. 28426828 10.1371/journal.pone.0175952 PMC5398566

[43] Siervo M, Montagnese C, Mathers JC, Soroka KR, Stephan BC, Wells JC Sugar consumption and global prevalence of obesity and hypertension: an ecological analysis. Public Health Nutr. 2014;17:587–96. 23414749 10.1017/S1368980013000141 PMC10282320

[44] Luger M, Lafontan M, Bes-Rastrollo M, Winzer E, Yumuk V, Farpour-Lambert N Sugar-sweetened beverages and weight gain in children and adults: a systematic review from 2013 to 2015 and a comparison with previous studies. Obesity Facts. 2017;10:674–93. 29237159 10.1159/000484566 PMC5836186

[45] Cornelsen L, Green R, Turner R, Dangour AD, Shankar B, Mazzocchi M, Smith RD What happens to patterns of food consumption when food prices change? Evidence from a systematic review and meta-analysis of food price elasticities globally. Health Econ. 2015;24:1548–59. 25236930 10.1002/hec.3107

[46] Muhammad A, D'Souza A, Meade B, Micha R, Mozaffarian D How income and food prices influence global dietary intakes by age and sex: evidence from 164 countries. BMJ Global Health. 2017;2(3):e000184. 10.1136/bmjgh-2016-000184 PMC5717967 29225943

[47] Ruel MT, Alderman H Nutrition-sensitive interventions and programmes: how can they help to accelerate progress in improving maternal and child nutrition?. Lancet North Am Ed. 2013;382:536–51. 10.1016/S0140-6736(13)60843-0 23746780

