# Supplementary material for: The Relative Caloric Prices of Healthy and Unhealthy Foods Differ Systematically across Income Levels and Continents
Source: J Nutr. 2019 Jul 23;149(11):2020–33. doi: 10.1093/jn/nxz158 (PMC6825829; doi:10.1093/jn/nxz158)
Supplement: nxz158_Supplemental_File [file nxz158_supplemental_file.pdf]

Supplementary data

Supplemental Table S1. List of standard definition food and beverage products in the 2011 International Comparison Program

Food group	ICP product name
cassava	Whole Cassava
	Cassava - Manioc - Yuka
maize	Maize Flour Yellow
	Maizena
	White Maize grains
	Yellow Broken Maize Grains
	Yellow Maize Grains
	Yellow Maize Grains, Branless
	Breakfast corn cereal
	Corn
	Corn tortilla
	Corn/Maize Flour, loose
	Cornflakes [Specified brand 1]
	Cornflakes [Specified brand 1], smaller package
	Cornflakes [Specified brand]
	Maize
	Maize
	Maize Flour White
	Maize Flour White
	Maize semolina
	Tinned sweet corn/Maize
	Yellow maize flour
millet	Couscous (millet)
	Millet Flour
	Millet Whole Grain
	Bajra Flour
	Millet, Sorghum
oats	Oats, rolled
rice	Long-grained rice
	Basmati Rice
	Beaten rice, Chira
	Brown rice - Family Pack
	Brown rice - small pack
	Coarse #2
	Coarse #3
	Coarse #5

Supplementary data

	Coarse #6
	Fresh rice noodles
	Glutinous Rice
	Jasmine Rice
	Long grain rice - Family Pack
	Long grain rice - Non-Parboiled
	Long grain rice - Parboiled
	Long grain rice - loose
	Premium rice #1
	Premium rice #2
	Premium rice #3
	Rice [Specified brand]
	Rice flour
	Short-grained rice
	Short-grained rice - prepacked
	Thailand Rice
	White rice #1
	White rice #10
	White rice #3
	White rice #4
	White rice #5
	White rice #6
	White rice #7
	White rice #9
	White rice, 20% broken
	White rice, 25% broken
	White rice, Medium Grain
	White rice, medium grain - prepacked
sorghum	Sorghum Red Grains
	Sorghum White Grains
wheat	Flatbread
	Lasagne (sheets)
	Round bread
	Shells
	Sliced brown bread
	Sweet Bread
	Baguette
	Breakfast wheat cereal
	Dried Noodles
	Egg noodles
	Flat (Iranian) Bread
	Hard Loose Bulgur
	Instant Noodles

Supplementary data

	Instant noodles
	Kaak (Bakssamat)
	Kiln Bread
	Lasagne
	Macaroni
	Pita bread
	Pita bread
	Roll
	Roll
	Roll or bun, loose
	Roll or bun, prepacked
	Sattu
	Semolina, Suji
	Short past with eggs
	Short pasta
	Sliced White bread
	Soft/ Loose Bulgur
	Spaghetti
	Spaghetti, with eggs
	Toast petit beurre brown crunchy
	Vermicelli (Angel Hair)
	Vermicelli (angel hair), with eggs
	Wheat Semolina (Suji)
	Wheat flour, loose
	Wheat flour, not self-rising
	White bread
	White bread, unsliced loaf
	White wheat
	Whole wheat bread
	Wholemeal flour, Atta
yam	Malanga / yautia / tannia / tannier / macabo
	Taro
potato	Brown Potatoes
	Frozen chipped potatoes
	White potato
Dark green leafy vegetables	Bean Leaves
	Cassava Leaves
	Pumpkin leaves
	Rape Leaves
	Spinach
	Sweet Potato Leaves
	Taro Leaves
	Lettuce

Supplementary data

	Spinach
	Spinach Chinese
	Water Spinach
eggs	Eggs, traditional production
	Chicken egg, 1
	Chicken eggs, 10, loose
	Large size chicken eggs
	Medium size chicken eggs
	Salted duck egg
	White or brown chicken eggs, Small size [domestic]
Fish/seafood	Anchovy
	Bream
	Capitaine
	Catfish
	Dried Machoiron
	Dried bonga
	Dried sardines
	Dried small fish
	Fresh Small Sardines
	Frozen Capitaine in Sea Water
	Frozen Nile Perch
	Frozen Sea-bream
	Frozen Shrimps
	Frozen Whiting
	Giant shrimps
	Grouper
	Lobster, chilled
	Mackerel in vegetable oil
	Nile Perch
	Red Snapper
	Red mullet
	Sardines in tomato sauce
	Smoked carp
	Smoked kapenta
	Smoked kingfish
	Smoked mboto
	Smoked shrimps/prawns
	Sole fish
	Tuna
	Tuna in vegetable oil
	Black Pomfret
	Canned Tuna/Water
	Canned mackerel fillet in vegetable oil

Supplementary data

Canned sardine with skin
Canned sardines with skin, in tomato sauce
Canned tuna without skin
Carp
Catfish
Caviar
Cod (Gadus morhua)
Dried Shrimp
Emperor fish
Fishball
Giant Shrimp
Grouper (Hamour) fish
Hake fillet
Mackerel, un-cleaned
Maigre
Maigre fillet
Mud Crab
Mullet
Prawn/Shrimp, medium
Prawn/Shrimp, small
Processed shrimp
Red porgy
Red porgy fillet
Red snapper
Salted & semi-dried fish
Salted dry cod
Sardines
Sea Bass
Sea Crab
Sea Lobster
Shrimps
Small fresh fish
Smoked fish
Smoked salmon
Sole
Spanish Mackerel
Squid
Squid, small
Surubi fillet
Tilapia
Tilapia fillet
Tuna
Tuna Steak
Tuna fish fresh
Tuna steaks

	White Pomfret
	Whole Shrimps
	Zubaida Fish
Fortified infant cereal	Baby food
	Domestic Corn Based Baby Food
	Rice Based Baby Food
	Wheat Based Baby Food
	Baby cereals
	Baby food
juice	Ginger juice (fresh)
	Guava juice
	Lime juice
	Mango juice
	Orange Drink
	Pineapple juice
	Pineapple juice freshly squeezed
	Tomato juice
	Apple
	Apple juice
	Fruit juice, not from concentrate, ready to drink
	Fruit nectars (single flavor)
	Instant fruit-juice flavored drink, powder
	Orange
	Orange
	Orange juice
	Orange juice (fresh)
	Orange juice - nectar
	Powdered Juice Mix [Specified brand]
milk	Powdered milk
	Buffalo milk, not pasteurized
	Buffalo milk, pasteurized
	Fresh Milk, 0% (import)
	Fresh Milk, 1.5-2.5%
	Fresh Milk, 3-4%
	Fresh Milk, 3-4%
	Milk, low - fat , pasteurized in plastic bag
	Milk, low-fat, Pasteurized
	Milk, low-fat, UHT
	Milk, not pasteurized
	Milk, powdered
	Milk, un-skimmed Pasteurized
	Milk, un-skimmed UHT

	Powdered milk, box
	Yoghurt, with flavor
nuts	Caramel groundnuts
	Cashew nuts
	Macadamia nuts
	Natural Groundnuts
	Roasted groundnuts
	Almonds, Unhusked
	Cashew
	Domestic Peanuts
	Dried almonds
	Hazelnuts
	Imported Peanuts
	Peanuts in shell
	Roasted groundnuts/peanuts
	Walnuts
	Watermelon seeds
Other dairy	Crème fraîche
	Fresh cheese edam
	Fresh cheese emmental
	Liquid Yoghurt
	Sour (clotted) milk
	Yoghurt with natural fruits
	Cheese spread
	Cheese, Camembert Type
	Cheese, Cheddar
	Cheese, Cottage (halloumi)
	Cheese, Feta
	Cheese, Gouda Type
	Cheese, Haloumi
	Cheese, Kashkaval
	Cheese, Mozzarella
	Cheese, mozzarella type
	Cheese, processed
	Coffee whitener [Specified brand 1], jar
	Coffee whitener [Specified brand 1], packet
	Cream cheese
	Fresh cheese
	Fresh wet cheese
	Labneh
	Light whipping or whipping
	Local cheese
	Local curd
	Local hard, dry cheese

Supplementary data

	Powdered milk, in bag or box
	Sour cream
	Yoghurt drink
	Yoghurt, fruit
	Yoghurt, plain
	Yogurt drink
Other fruit	Banana, short fionger length
	Clementine
	Dried plums
	Grapes, red
	Green Plantain
	Passion fruit
	Apple, Red Delicious
	Apple, Typical Local Variety
	Banana, Standard
	Dark raisins
	Domestic Cherries
	Domestic Figs
	Domestic Grapes
	Domestic Pears
	Domestic Plums
	Domestic Pomegranate
	Domestic Red Dates
	Domestic Strawberries
	Domestic Dates
	Dried dates
	Figs
	Grapefruit
	Grapes, green
	Grapes, violet, with seed
	Imported Cherries
	Imported Coconut
	Imported Dates
	Imported Figs
	Imported Grapes
	Imported Kiwi
	Imported Pears, Premium
	Imported Plums
	Imported Pomegranate
	Imported Red Dates
	Lemon
	Lime
	Melon
	Melon, Honeydew

Supplementary data

	Orange
	Passion fruit
	Pineapple
	Tamarind
	Tamarind, preserved
	Tinned pineapple
	Watermelon
Other vegetables	Beetroots
	Black Olives
	Broccoli
	Celery
	Chilies
	Chives
	Dried Okra
	Fresh Okra
	Gherkins
	Green Asparagus
	Mushrooms
	Peeled Tomatoes
	Radish
	Round Onions, red
	Sorrel Leaves
	Spring Onions
	Turnips
	Avocado
	Beetroot
	Cauliflower
	Celery
	Chilies (Long)
	Chilies, dried
	Cucumber
	Cucumber Pickles
	Domestic Beetroot
	Domestic Garlic
	Domestic Green bell peppers
	Domestic Okra
	Domestic Round red radish
	Domestic Tomatoes
	Domestic White onion
	Domestic Zucchini
	Eggplant (aubergine)
	Garlic (White)
	Ginger (Mature)
	Green Olives (with stones)

Supplementary data

	Green cabbage
	Imported Beetroot
	Imported Garlic
	Imported Okra
	Imported Tomatoes
	Imported White onion
	Imported Zucchini
	Mushrooms, dried
	Okra
	Onion
	Plum tomatoes
	Radish, white
	Ripe(black) Olives
	Ripe(black) Olives, can or unpackaged
	Round tomato, loose
	Tinned Button Mushrooms
	Tomato paste (Large)
	Tomato paste (Small)
	Vegetarian vegetable soup
	Zucchini
Oils/fats	Maize oil
	Sesame oil
	Corn oil
	Liquid Tahina
	Maize oil
	Mustard oil
	Olive Oil
	Olive oil
	Olive oil, standard
	Peanut butter
	Peanut butter
	Peanut oil
	Pure Sesame Oil
	Sesame oil or oil Alserg
	Soybean oil
	Sunflower oil
	Tahina
	Vegetable oil
	Butter, sold loose
	Ghee
	Palm oil unrefined
	Animal Fats
	Butter, unsalted
	Coconut oil

Supplementary data

	Ghee
	Ghee, cow/buffalo
	Ghee, vegetable
	Margarine, regular fat
	Palm oil
	Palm oil
	Salted Butter
pulses	Broad Beans
	Green Beans
	Packed Peas
	Peas
	Peas
	Pigeon peas
	Spotted beans
	Bean Curd - Tofu
	Dhal, Khesari
	Dhal, Musur
	Dhal, Split Peas
	Domestic Broad beans (Pulses)
	Domestic Green beans (Pulses)
	Domestic Peas
	Dried black beans
	Dried red beans
	Dried white beans
	Green beans
	Green/Mung Beans, dried
	Imported Broad beans (Pulses)
	Imported Green beans (Pulses)
	Imported Peas
	Imported Peas
	Lentils, Dry
	Moong dahl, loose
	Peas, Tinned
	Tinned green peas
	Tinned white beans in tomato sauce
Processed red meat	Beef ham
	Sausage
	Breakfast sausage, chicken
	Canned beef, chunks
	Mortadella, loose
	Mortadella, prepacked
	Pork and beef sausages
	Pork ham, pressed

Supplementary data

	Pork ham, pressed, bulk or loose
	Sliced ham, pork
Unprocessed red meat	Beef Feet/Trotters (Uncleaned)
	Beef Merguez (spiced)
	Beef prepacked
	Beef without bones
	Lamb
	Live Goat
	Live Sheep
	Mutton Tripe
	Mutton chop
	Oxtail
	Pork meat
	Sirloin Steak
	Veal without offal
	100% Beef, minced
	Bacon, pork
	Bacon, smoked
	Beef liver
	Beef with bones
	Beef, Center brisket
	Beef, Fillet
	Beef, Fillet, frozen
	Beef, Rump steak
	Beef, for stew or curry
	Beef, with bones, non-specific cut
	Beef, without bones, non-specific cut
	Buffalo, without bones, non-specific cut
	Corned beef
	Domestic Fillet Steak(excluding round &sirloin)
	Domestic Ground mutton (Fresh)
	Fillet (Round or sirloin)
	Flank or skirt beef, for shredding
	Goat leg
	Goat mixed cut/with bones (non-refrigerated)
	Goat, boneless. Non-specific cut
	Ground beef (Frozen)
	Imported Fillet Steak(excluding round &sirloin)
	Imported Ground Mutton (Fresh)
	Lamb (Fresh) with bones , Non-specific cut
	Lamb (Fresh), boneless, Non-specific cut
	Lamb chops
	Lamb whole leg
	Live lamb

Supplementary data

	Live mutton
	Mutton Liver (Chilled)
	Mutton Liver (Fresh)
	Mutton chops
	Mutton mixed cut
	Mutton with bones (Refrigerated) , Non-specific cut
	Mutton/goat liver
	Pork liver
	Pork loin, without bones
	Pork thigh, with bones
	Pork, fillet
	Pork, loin chop
	Pork, ribs
	Pork, shoulder
	Pork, with bones, non-specific cut
	Pork, without bones, non-specific cut
	Round steak
	Sirloin steak
	Veal breast (non-refrigerated), with bones
	Veal chops
	Veal with bones (Refrigerated)
	Veal, Boneless (Fresh unchilled)
	Veal, Boneless (Refrigerated)
	Veal, with bones
Salt-rich snacks	Cream crackers
	Potato chips
	Salted crackers
	Snack crackers
Soft drinks	Cola Drink
	Local Soft Drink
	Carbonated Soft Drink [Specified brands and model]
	Carbonated Soft Drink [Specified brands and model], small bottle
	Carbonated Soft Drink [Specified brands] (Large)
	Carbonated Soft Drink [Specified brands] (Small)
	Carbonated Soft Drink, can [Specified brands and model]
	Lemon-lime flavored Carbonated Soft Drink [Specified brand and model]
	Lemonade
	Soft drinks, small bottle
Sugar	Brown sugar cubes
	Packed Brown sugar
	Packed White sugar

Supplementary data

	Powdered Glucose
	Brown sugar
	White sugar
	White sugar, bulk
	White sugar, family size pack
	White sugar, granulated, cane
	White sugar, loose
Sugar-rich snacks	Chocolate biscuit
	Chocolate croissant
	Condensed milk sweetened
	Dark chocolate
	Doughnuts
	Ice cream cone
	Milk chocolate
	Regular chewing gum
	Simple cookie
	Sponge Cake
	Wafers
	All-butter croissant
	Baklawa
	Biscuit Assortment
	Butter biscuits
	Caramels and toffees
	Chinese cake/Moon cake
	Chocolate bar
	Chocolate bar [Specified brand]
	Chocolate cake (Individual serving)
	Chocolate cake (Whole)
	Cream biscuits
	Cup cakes
	Doughnuts
	Doughnuts
	Dulce de leche
	Flavored biscuits/cookies sweet
	Fruit cocktail, Canned
	Fruit drops (Hard candies)
	Fruit jellies
	Guava jam
	Guava jelly
	Hard candy, filled
	Ice cream, Cornetto-type
	Ice cream, Mixed
	Ice cream, packed
	Jam, high fruit content

Supplementary data

	Jam, low fruit content
	Katayef
	Knefeh
	Milk, condensed
	Muffin
	Natural honey, Mixed blossoms
	Orange marmalade
	Oranges jam
	Pineapple Jam
	Processed honey, pure
	Processed honey, pure
	Regular cake(multiple) with cream topping
	Sandwich biscuits/cookies
	Sandwich biscuits/cookies packaged
	Semi sweet baking chocolate
	Sponge Cake
	Strawberry/Apricot Jam
	Suckers lollipops
	Toffee
vA-rich fruits & vegetables	Dried apricots
	Large Mango (Grafted)
	Peppers
	Tinned peaches
	Apricots
	Bell pepper
	Buttercup squash
	Canned peach halves
	Carrots
	Domestic Apricots
	Domestic Carrots
	Domestic Guava
	Domestic Mango
	Domestic Red bell peppers
	Imported Apricots
	Imported Guava
	Imported Melon
	Imported Red bell peppers
	Mango
	Mixed Fruits in Syrup
	Papaya
	Peach
	Pumpkin
	Sweet Potatoes

Supplementary data

White meat	Chicken wings
	Duck - Dressed
	Gizzard
	Live Turkey
	Mix Frozen Chicken Parts
	Traditionally bred live chicken
	Turkey breast
	Canned chicken
	Chicken breast with skin and bones
	Chicken breast without skin
	Chicken drumsticks
	Chicken legs
	Chicken soup
	Chicken wings
	Chicken wings
	Chicken, non-specific cuts, frozen
	Chicken, non-specific cuts, not frozen
	Duck, whole
	Fresh whole chicken
	Live chicken
	Native house chicken
	Poultry sausages (chicken or turkey)
	Whole chicken
	Whole chicken (Frozen)
	Whole chicken - Broiler

Source: World Bank International Comparison Program technical directorate. For details, see the following publication: World Bank: Purchasing Power Parities and the Real Size of World Economies: A Comprehensive Report of the 2011 International Comparison Program. Washington, DC: World Bank; 2015.

Supplementary data

Supplemental Table S2. Survey frame characteristics for the 2011 International Comparison Program price data

		A. Survey coverage				B. Outlets selected										
		Geographical coverage	Population coverage	Urban and rural coverage	Prices adjusted for national average	Total		Large shops	Medium and small shops	Markets and street outlets	Bulk and discount shops	Specialized shops	Private service provider	Public or other service provider	Other
Economy			%			#	%	%	%	%	%	%	%	%	%
AFRICA															
Algeria	DZA	17 regions out of 48 total	35	Mostly urban	…	2,341	100	...	43	4	...	22	24	5	2
Angola	AGO	7 regions out of 18 total	66	Urban only	…	559	100	4	5	12	...	33	34	12	...
Benin	BEN	5 regions out of 6 total	12	Urban only	…	2,887	100	5	6	20	13	29	20	7	...
Botswana	BWA	14 regions out of 14 total	83	Urban and rural	…	491	100	10	14	...	...	42	13	1	20
Burkina Faso	BFA	Capital only	13	Urban only	…	125	100	2	15	9	1	29	35	9	...
Burundi	BDI	17 regions out of 17 total	94	Urban and rural	…	4,104	100	...	17	72	...	11	...	...	...
Cameroon	CMR	10 regions out of 10 total	97	Urban and rural	…	...	...	...	...	...	...	...	...	...	...
Cape Verde	CPV	5 regions out of 5 total	51	Urban and rural	…	510	100	3	30	53	1	7	2	2	2
Central African Republic	CAF	7 regions out of 7 total	85	Urban and rural	…	1,503	100	2	26	13	1	22	15	12	9
Chad	TCD	Capital only	8	Urban only	…	444	100	6	31	12	11	18	7	5	10
Comoros	COM	Capital only	6	Urban only	…	394	100	2	13	1	2	38	39	5	...
Congo	COG	12 regions out of 12 total	55	Urban only	…	1,774	100	...	16	3	1	38	36	4	2
Congo, Dem. Rep.	COD	11 regions out of 11 total	74	Urban and rural	…	2,288	100	1	14	6	6	35	35	2	1
Côte d'Ivoire	CIV	7 regions out of 10 total	82	Urban and rural	…	953	100	2	33	22	10	9	13	5	6

Supplementary data

	Djibouti	DJI	Capital only	56	Urban only	…	111	100	4	57	7	...	21	6	5	...
	Egypt, Arab Rep.[a]	EGY	11 regions out of 27 total	61	Urban and rural	…	6,000	100	3	22	22	...	47	2	4	...
	Equatorial Guinea	GNQ	...	...	...	…	...	...	...	...	...	...	...	...	...	...
	Ethiopia	ETH	...	...	...	…	...	...	...	...	...	...	...	...	...	...
	Gabon	GAB	2 regions out of 9 total	64	Urban only	…	461	100	6	9	9	7	27	14	12	16
	Gambia, The	GMB	7 regions out of 7 total	106	Urban and rural	…	787	100	4	37	48	...	6	3	2	...
	Ghana	GHA	10 regions out of 10 total	99	Urban and rural	…	1,940	100	...	37	37	...	6	13	6	1
	Guinea	GIN	...	...	...	…	...	...	...	...	...	...	...	...	...	...
	Guinea-Bissau	GNB	Capital only	24	Urban and rural	…	904	100	...	39	23	...	18	14	6	...
	Kenya	KEN	19 regions out of 19 total	13	Mostly urban	…	1,804	100	9	9	11	...	31	26	11	3
	Lesotho	LSO	10 regions out of 10 total	86	Urban and rural	…	855	100	9	23	...	...	54	2	12	...
	Liberia	LBR	5 regions out of 15 total	55	Urban and rural	…	123	100	12	24	39	...	11	11	3	...
	Madagascar	MDG	21 regions out of 22 total	96	Urban only	…	1,693	100	2	9	24	31	28	6	...	...
	Malawi	MWI	4 regions out of 4 total	89	Urban and rural	…	1,299	100	6	10	4	2	18	29	28	3
	Mali	MLI	9 regions out of 9 total	17	Urban only	…	9,376	100	2	40	11	7	27	9	4	...
	Mauritania	MRT	13 regions out of 13 total	93	Urban only	…	1,896	100	2	19	22	5	23	19	2	8
	Mauritius	MUS	10 regions out of 10 total	95	Urban and rural	…	391	100	7	29	3	...	45	11	5	...
	Morocco	MAR	9 regions out of 16 total	70	Urban and rural	…	2,507	100	3	24	12	...	40	18	2	1
	Mozambique	MOZ	4 regions out of 4 total	12	Urban only	…	828	100	7	12	8	...	62	9	2	
	Namibia	NAM	8 regions out of 13 total	34	Urban only	…	371	100	14	8	9	...	28	25	...	16

	Niger	NER	8 regions out of 8 total	...	Urban and rural	…	158	100	2	20	9	1	35	29	4	...
	Nigeria	NGA	…	...	Urban and rural	…	70	...	...	...	...	...	...	...	...	...
	Rwanda	RWA	11 regions out of 11 total	74	Urban and rural	…	946	100	...	19	3	...	76	1	1	...
	São Tomé and Principe	STP	26 regions out of 33 total	28	Urban and rural	…	841	100	1	25	2	2	32	26	8	4
	Senegal	SEN	5 regions out of 5 total	...	Urban only	…	1,533	100	...	3	1	11	27	36	17	5
	Seychelles	SYC	5 regions out of 5 total	105	Urban only	…	176	100	9	14	2	...	47	15	13	...
	Sierra Leone	SLE	4 regions out of 4 total	32	Urban and rural	…	...	...	...	...	...	...	...	...	...	...
	South Africa	ZAF	3 regions out of 3 total	...	Urban only	…	537	100	46	23	...	...	31	...	...	...
	Sudan[b]	SDN	15 regions out of 15 total	73	Urban and rural	…	889	100	14	22	21	6	13	11	9	4
	Swaziland	SWZ	4 regions out of 4 total	...	Urban and rural	…	155	100	15	26	16	...	32	8	3	...
	Tanzania	TZA	14 regions out of 14 total	...	Urban and rural	…	380	100	4	19	22	...	18	19	18	...
	Togo	TGO	5 regions out of 5 total	38	Urban only	…	1,282	100	1	3	4	...	47	11	4	30
	Tunisia	TUN	Capital only	...	Urban and rural	…	369	100	5	22	4	8	9	33	19	...
	Uganda	UGA	5 regions out of 5 total	95	Urban and rural	…	5,649	100	1	11	7	...	39	42	...	...
	Zambia	ZMB	53 districts out of 73 total	...	Urban only	…	...	...	...	...	...	...	...	...	...	...
	Zimbabwe	ZWE	10 regions out of 10 total	91	Urban only	…	9,547	100	43	8	8	...	35	3	1	2

**ASIA AND THE PACIFIC**																
	Bangladesh	BGD	23 areas covered	...	Urban and rural	...	...	...	...	...	...	...	...	...	...	...
	Bhutan	BTN	22 areas covered	...	Urban and rural	...	752	100	2	54	6	...	4	27	2	5

Supplementary data

	Brunei Darussalam	BRN	4 areas covered	...	Urban only	...	298	100	4	3	2	...	48	39	3	1
	Cambodia	KHM	18 out of 24 areas covered	...	Urban and rural	...	3,236	100	3	23	50	...	4	17	3	...
	China	CHN	30 areas covered	...	Urban and rural	...	585	100	8	19	7	...	22	31	11	2
	Fiji	FJI	3 areas covered	...	Urban only	...	688	100	14	8	10	...	24	14	25	5
	Hong Kong SAR, China	HKG	Entire territory covered	...	Urban only	...	2,903	100	6	9	16	...	35	29	5	...
	India	IND	31 areas covered	...	Urban and rural	...	...	...	...	...	...	...	...	...	...	...
	Indonesia	IDN	33 areas covered	...	Urban and rural	...	2,645	100	3	12	56	...	12	11	5	1
	Lao PDR	LAO	12 areas covered	...	Urban and rural	...	1,398	100	...	8	53	1	6	22	9	1
	Macao SAR, China	MAC	Entire territory covered	...	Urban only	...	...	100	4	8	7	...	46	33	2	...
	Malaysia	MYS	13 areas covered	...	Urban and rural	...	13,982	100	13	24	21	...	30	10	2	...
	Maldives	MDV	5 areas covered	...	Urban and rural	...	475	100	1	57	6	...	9	19	2	6
	Mongolia	MNG	22 areas covered	...	...	...	1,664	100	5	33	5	...	6	26	15	10
	Myanmar	MMR	17 areas covered	...	Urban and rural	...	4,328	100	1	11	52	...	16	14	5	1
	Nepal	NPL	30 areas covered	...	Urban and rural	...	4,577	100	1	18	21	1	29	26	3	1
	Pakistan	PAK	31 areas covered	...	Mostly urban	...	21,896	100	3	1	3	...	39	40	7	7
	Philippines	PHL	17 areas covered	...	Urban and rural	...	6,351	100	6	9	31	...	30	18	3	3
	Singapore	SGP	Entire territory covered	...	Urban only	...	...	...	...	...	...	...	...	...	...	...
	Sri Lanka	LKA	23 areas covered	...	Urban and rural	...	2,806	100	4	65	13	...	7	9	1	1
	Taiwan, China	TWN	4 areas covered	...	Urban only	...	...	100	2	2	13	...	47	26	9	1
	Thailand	THA	25 areas covered	...	Urban and rural	...	2,705	100	4	10	16	1	34	25	10	...

Supplementary data

	Vietnam	VNM	17 areas covered	...	Urban and rural	...	306	100	12	8	37	...	17	15	11	...
**COMMONWEALTH OF INDEPENDENT STATES**																
	Armenia	ARM	3 regions out of 11 total	...	Urban only	...	1,177	100	7	14	4	...	33	36	6	...
	Azerbaijan	AZE	54 regions out of 67 total	...	Urban only	...	3,271	100	8	26	2	...	5	40	19	...
	Belarus	BLR	6 regions out of 7 total	67	Urban only	...	473	100	8	21	10	1	21	17	7	15
	Kazakhstan	KAZ	16 regions out of 16 total	...	Urban only	...	4,682	100	5	10	4	...	32	41	8	...
	Kyrgyzstan	KGZ	8 regions out of 9 total	...	Urban only	...	299	100	5	16	11	...	21	43	4	...
	Moldova	MDA	8 larger cities	...	Urban only	...	927	100	6	11	3	...	32	21	14	13
	Russian Federation[c]	RUT	capital city and 1 region	...	Urban only	...	861	100	9	10	6	2	35	17	6	15
	Tajikistan	TJK	4 regions out of 5 total	...	Urban and rural	...	306	100	7	19	16	...	18	16	14	10
	Ukraine	UKR	capital city	...	Urban only	...	1,107	100	2	8	1	...	58	28	3	...
**EUROSTAT-OECD[d]**																
	Albania	ALB	Capital city	...	Urban only	...	...	100	5	37	6	...	33	19	...	...
	Australia	AUS	10 capital cities	...	Urban only		...	...	...	...	...	...	...	...	...	...
	Austria	AUT	Capital city	...	Urban only	...	...	100	8	17	4	3	45	18	...	5
	Belgium	BEL	10 cities	...	...	...	...	100	13	35	1	3	30	14	2	2
	Bosnia and Herzegovina	BIH	Capital city	...	Urban only	...	...	100	1	48	3	...	31	13	3	1
	Bulgaria	BGR	Capital city	...	Urban only	Yes	...	100	6	34	3	2	41	13	...	1
	Canada	CAN	Main cities	...	Urban only	..	...	...	...	...	...	...	...	...	...	...
	Chile	CHL	Capital city or main cities	...	...	...	...	...	...	...	...	...	...	...	...	...
	Croatia	HRV	Capital city	...	Urban only	...	...	100	8	30	1	...	38	18	3	2
	Cyprus	CYP	Capital city	...	Urban only	...	...	100	12	21	...	4	34	16	3	10
	Czech Republic	CZE	Capital city	...	Urban only	Yes	...	100	35	29	...	3	17	12	...	4
	Denmark	DNK	Capital city	...	Urban only	...	...	100	3	27	...	9	37	10	...	14

Supplementary data

	Estonia	EST	Capital city	...	Urban only	...	…	100	12	36	1	1	33	14	3	...
	Finland	FIN	Capital city	...	Urban only	...	…	100	13	27	...	4	29	20	1	6
	France	FRA	Capital city	...	Urban only	Yes	…	100	8	41	1	2	23	21	...	4
	Germany	DEU	2 cities	...	...	...	…	100	12	23	...	4	34	14	3	10
	Greece	GRC	Capital city	...	Urban only	...	…	100	6	36	1	1	38	17	...	1
	Hungary	HUN	Capital city	...	Urban only	Yes	…	100	4	34	4	2	35	18	1	2
	Iceland	ISL	Capital city	...	Urban only	...	…	100	9	18	...	5	32	18	1	17
	Ireland	IRL	Capital city	...	Urban only	...	…	100	16	38	...	...	25	18	1	2
	Israel	ISR	Main cities	...	Urban only		…	...	...	...	...	...	...	...	...	...
	Italy	ITA	Capital city	...	Urban only	Yes	…	100	11	39	1	2	27	15	...	5
	Japan	JPN	Capital city	...	Urban only	Yes	…	...	...	...	...	...	...	...	...	...
	Korea, Rep.	KOR	Capital city + 2 other big cities	...	Urban only	..	…	...	...	...	...	...	...	...	...	...
	Latvia	LVA	Capital city	...	Urban only	...	…	100	12	33	2	1	26	17	2	7
	Lithuania	LTU	Capital city	...	Urban only	...	…	100	1	30	2	...	45	17	...	5
	Luxembourg	LUX	Capital city	...	Urban only	...	…	100	...	44	...	1	30	19	1	5
	Macedonia, FYR	MKD	Capital city	...	Urban only	...	…	100	...	33	2	...	45	20	...	...
	Malta	MLT	Capital city	...	Urban only	...	…	100	3	35	...	...	37	24	1	...
	Mexico	MEX	Main cities	...	...	...	…	...	...	...	...	...	...	...	...	...
	Montenegro	MNE	Capital city	...	Urban only	...	…	100	...	36	1	...	55	8	...	...
	Netherlands	NLD	Capital city	...	Urban only	Yes	…	100	21	31	1	1	31	14	...	1
	New Zealand	NZL	Capital city	...	...	..	…	...	...	...	...	...	...	...	...	...
	Norway	NOR	Capital city	...	Urban only	...	…	100	...	38	...	15	28	16	...	3
	Poland	POL	Capital city	...	Urban only	Yes	…	100	6	34	3	1	33	18	2	3
	Portugal	PRT	Capital city	...	Urban only	Yes	…	100	10	33	1	1	33	20	1	1
	Romania	ROU	8 cities	...	...	...	…	100	2	42	1	1	33	18	1	2
	Russian Federation [c]	RUS	2 regions out of 2 total	...	Urban only	...	…	...	...	...	...	...	...	...	...	...
	Serbia	SRB	Capital city	...	Urban only	...	…	100	1	49	5	1	30	11	2	1
	Slovakia	SVK	Capital city	...	Urban only	...	…	100	15	38	1	...	28	17	...	1
	Slovenia	SVN	Capital city	...	Urban only	...	…	100	8	40	...	3	25	15	1	8
	Spain	ESP	Capital city	...	Urban only	Yes	…	100	6	30	5	1	37	19	1	1
	Sweden	SWE	Capital city	...	Urban only	Yes	…	100	4	58	...	...	22	12	1	3
	Switzerland	CHE	Capital city	...	Urban only	...	…	100	12	25	...	5	33	11	3	11
	Turkey	TUR	Capital city	...	Urban only	Yes	…	100	17	31	...	1	35	15	1	...

Supplementary data

	United Kingdom	GBR	Capital city	...	Urban only	Yes	…	100	18	33	...	...	32	10	2	5
	United States	USA	All States	...	Urban only		…	...	...	...	...	...	...	...	...	...
**LATIN AMERICA**[e]															
	Bolivia	BOL	10 cities	49	Urban only	...	...	100	13	14	29	9	13	13	9	...
	Brazil	BRA	4 cities	43	Urban only	...	...	100	12	12	24	6	13	12	12	9
	Colombia	COL	...	...	...	...	...	...	...	...	...	...	...	...	...	...
	Costa Rica	CRI	4 cities	55	Urban only	...	...	100	18	18	18	...	18	18	5	5
	Cuba	CUB	...	...	...	...	...	...	...	...	...	...	...	...	...	...
	Dominican Republic	DOM	10 cities	41	Urban only	...	...	100	15	21	17	4	20	17	4	2
	Ecuador	ECU	2 cities	32	Urban only	...	...	100	18	18	18	...	19	18	...	9
	El Salvador	SLV	23 cities	33	Urban only	...	...	100	14	18	28	...	19	21	...	...
	Guatemala	GTM	...	...	...	...	...	...	...	...	...	...	...	...	...	...
	Haiti	HTI	…	...	...	...	...	100	17	...	50	...	...	16	...	17
	Honduras	HND	2 cities	22	Urban only	...	...	100	33	...	...	...	33	34	...	...
	Nicaragua	NIC	9 cities	57	Urban only	...	...	100	22	22	...	...	22	22	12	...
	Panama	PAN	...	...	...	...	...	...	...	...	...	...	...	...	...	...
	Paraguay	PRY	Capital city	8	Urban only	...	...	100	14	14	29	...	14	15	14	...
	Peru	PER	Capital city	31	Urban only	...	...	100	14	14	29	...	14	15	14	...
	Uruguay	URY	1 city	...	...	...	...	100	20	20	20	...	20	20	...	...
	Venezuela, RB	VEN	2 cities	12	Urban only	...	...	100	14	14	14	7	14	16	14	7
**CARIBBEAN**															
	Anguilla	AIA	14 cities	89	Urban only	...	119	100	5	10	...	2	33	47	3	...
	Antigua and Barbuda	ATG	Capital city and surrounding area	62	Urban and rural	...	125	100	3	...	...	...	2	90	5	...
	Aruba	ABW	8 cities	92	Urban only	...	186	100	4	-	6	-	34	50	5	...
	Bahamas, The	BHS	1 city	67	Urban only	...	137	100	6	24	...	...	31	35	4	...
	Barbados	BRB	11 cities	99	Urban and rural	...	293	100	2	6	1	...	26	64	1	...
	Belize	BLZ	...	...	...	...	...	...	...	...	...	...	...	...	...	...

	Bermuda	BMU	...	...	...	...	...	...	...	...	...	...	...	...	...	...
	Bonaire	BON	...	...	...	...	...	...	...	...	...	...	...	...	...	...
	Cayman Islands	CYM	4 cities	89	Urban only	...	173	100	6	...	1	1	31	58	3	...
	Curaçao	CUW	Capital city	99	Urban only	...	299	100	4	8	...	1	50	33	4	...
	Dominica	DMA	...	...	...	...	...	...	...	...	...	...	...	...	...	...
	Grenada	GRD	2 cities	61	Urban only	...	189	100	6	11	...	...	56	20	7	...
	Jamaica	JAM	15 cities	98	Urban and rural	...	139	100	16	14	8	1	14	21	22	4
	Montserrat	MSR	3 cities	96	Urban only	...	206	100	6	34	4	1	11	43	1	...
	St. Kitts and Nevis	KNA	...	...	...	...	...	...	...	...	...	...	...	...	...	...
	St. Lucia	LCA	...	...	...	...	...	...	...	...	...	...	...	...	...	...
	St. Vincent and the Grenadines	VCT	15 cities	51	Urban and rural	...	131	100	11	2	4	...	28	50	5	...
	Sint Maarten	SXM	7 cities	...	...	...	81	100	10	...	...	1	29	53	6	1
	Suriname	SUR	...	...	...	...	...	...	...	...	...	...	...	...	...	...
	Trinidad and Tobago	TTO	...	...	...	...	...	...	...	...	...	...	...	...	...	...
	Turks and Caicos Islands	TCA	...	...	...	...	...	...	...	...	...	...	...	...	...	...
	Virgin Islands, British	VGB	...	...	...	...	...	...	...	...	...	...	...	...	...	...
**WESTERN ASIA**																
	Bahrain	BHR	5 regions out of 5 total	100	Urban only	...	494	100	2	3	1	1	45	47	1	...
	Egypt, Arab Rep.[a]	EGZ	11 regions out of 27 total	61	Urban and rural	...	6,000	100	3	22	22	...	47	2	4	...
	Iraq	IRQ	8 regions out of 18 total	44	Urban only	...	256	100	25	...	...	...	...	25	25	25
	Jordan	JOR	12 regions out of 12 total	83	Urban only	...	3,065	100	3	13	13	...	28	42	1	...
	Kuwait	KWT	6 regions out of 6 total	100	Urban only	...	36	100	17	17	16	...	16	17	17	...

Supplementary data

	Oman	OMN	8 regions out of 11 total	86	Urban only	...	1,241	100	3	1	5	...	88	3	...	...
	Palestinian Territory	PSE	5 regions out of 16 total	37	Urban only	...	2,276	100	3	24	1	...	47	24	1	...
	Qatar	QAT	3 regions out of 3 total	79	Urban only	...	556	100	4	40	2	...	19	28	2	5
	Saudi Arabia	SAU	5 regions out of 13 total	73	Urban only	...	1,447	100	...	100	...	...	...	...	...	...
	Sudan[b]	SDO	15 regions out of 15 total	73	Urban and rural	...	889	100	14	22	20	6	13	12	9	4
	United Arab Emirates	ARE	…	...	...	...	1,603	100	2	8	5	5	44	23	3	10
	Yemen	YEM	3 regions out of 21 total	16	Urban and rural	...	424	100	4	60	6	...	...	22	8	...

… Data not available.
a. Egypt participated in both the Africa and Western Asia comparisons.
b. Sudan participated in both the Africa and Western Asia comparisons.
c. The Russian Federation participated in both the CIS and Eurostat-OECD comparisons.
d. The outlet type shares are approximates, after mapping the Eurostat/OECD shop type classification to the ICP shop type classifications.
e. Latin American economies' outlet type shares are approximations.

Source: World Bank International Comparison Program technical directorate. For details, see the following publication: World Bank: Purchasing Power Parities and the Real Size of World Economies: A Comprehensive Report of the 2011 International Comparison Program. Washington, DC: World Bank; 2015.

Supplementary data

Supplemental Table S3. The cost of 1000 calories of a country-specific basket of starchy staples in 176 countries, by World Bank income levels and major regions (population-weighted estimates in 2011 international dollars)[1]

	N (countries)	Cost per 1000 calories
All countries	176	$0.75
a. High income	64	$1.16
b. Upper middle	44	$0.82
c. Lower middle	41	$0.55
d. Low income	27	$0.42
a. Europe	39	$1.03
b. North America & Australasia	6	$1.44
c. Latin America	38	$1.18
d. Middle East & North Africa	18	$0.68
e. Central Asia	6	$0.73
f. China	1	$0.72
g. Rest of East Asia	5	$1.00
h. South-East Asia	10	$0.53
i. India	1	$0.58
j. Rest of South Asia	6	$0.53
k. Eastern & Southern Africa	19	$0.50
l. Western & Central Africa	27	$0.36

[1] Authors' estimates from ICP price data. The index of starchy staple prices in each country was constructed as follows. First, prices per 1000 calories of each starchy food in local currency units were converted to international dollars using the purchasing power parity conversion factor for foods and beverages. Then the weights on each of nine starchy food groups (rice, maize, wheat, millet, oats, sorghum, cassava, yam, potato) were applied to the median food price in each country for each of these nine starchy food groups.

Supplementary data

Table S4. Robust regressions of 7-day recall estimates of consumption prevalence of nine different food groups among women 15-49y against corresponding Relative Caloric Prices (RCPs) and GDP per capita (both in logs)[1]

	Vitamin A-rich fruit/ vegetables	Dark green leafy vegetables	Other fruit	Pulses & nuts	Dairy	Eggs	Fish	Meat	Oils/fats	Sweets
Own price (RCP), log	0.02	-0.08	-0.09	0.04	-0.13	-0.13*	-0.21	0.04	-0.14*	-0.16*
	(-0.13,0.16)	(-0.23,0.07)	-(0.22,0.04)	(-0.22,0.30)	(-0.32,0.07)	(-0.25,-0.02)	(-0.50,0.08)	(-0.17,0.25)	(-0.28,-0.00)	(-0.29,-0.03)
GDP per capita, log	0.07	-0.05	0.12**	0.05	0.18*	0.12**	-0.06	0.18***	0.10*	0.13***
	(-0.02,0.15)	-0.15,0.05)	(0.04,0.20)	(-0.07,0.16)	(0.04,0.31)	(0.04,0.20)	(-0.21,0.09)	(0.09,0.27)	(0.01,0.20)	(0.06,0.19)
R^2	0.12	0.16	0.39	0.04	0.35	0.58	0.13	0.46	0.29	0.57
N (countries)	24	23	25	26	25	26	24	25	24	25
Consumption prevalence (%)	39.5%	45.0%	38.9%	39.1%	30.6%	28.2%	38.4%	37.4%	55.6%	31.6%
Mean CPR	6.3	14.7	3.2	1.5	6.5	7.4	7.3	3.3	1.0	2.3

[1] All values are βs (95% CIs) derived via the robust regression command in STATA 14 (*rreg*) which downweighs outlying observations, but results are qualitatively similar with the least squares estimator. Consumption prevalence refers to the percentage of women who reported consuming the food in the past 7 days. RCP is the relative caloric price, as defined in the main text. *,**,*** Significant differences: ***P<0.001, **P<0.01,*P<0.05.

Supplementary data

Supplemental Table S5. Cross-country robust regression estimates of the associations between overweight/obesity prevalence among adults 15-59 and the prices of various sugar-rich, fat-rich and salt-rich foods in models that adjust for relative caloric prices (RCPs) for multiple food groups[1]

	Model 1	Model 2	Model 3	Model 4
Sugar RCP, log	-4.75***	-5.45***		
	(-6.66,-2.83)	(-7.58,-3.31)		
Soft RCP, log			-7.43***	-9.81***
			(-10.69,-4.16)	(-13.30,-6.33)
Fats/oils RCP, log		-0.22		1.4
		(-3.05,2.61)		(-1.66,4.47)
Potato chip RCP, log		3.51*		4.75**
		(0.56,6.45)		(1.94,7.55)
R^2	0.84	0.84	0.81	0.87
N	128	115	127	114

[1] All values are βs (95% CIs) derived via the robust regression command in STATA 14 (*rreg*) which downweighs outlying observations, although results are qualitatively similar with the least squares estimator. RCP is the relative caloric price, as defined in the main text. All models are adjusted for GDP per capita, the urban population share, the 15+years female labor force participation rate, and the 15+years female literacy rate, all specified in logs, but models 2 and 4 include RCPs for fats/oils and potato chips.
*,**,*** Significant differences: ***P<0.001, **P<0.01,*P<0.05.

Supplementary data

Supplemental Table S6. Cross-country least squares regression estimates of the associations between overweight prevalence among adults 25 years and older and the relative caloric prices (RCPs) of various unhealthy foods in unadjusted and adjusted models[1]

	Sugar RCP, logged	Soft drink RCP, logged	Oil/fat RCP, logged	Potato chip RCP, logged
Unadjusted model	-11.87***	-18.65***	-11.99***	-11.22***
	(-15.42,-8.32)	(-22.57,-14.73)	(-17.54,-6.44)	(-16.12,-6.32)
R^2	0.28	0.44	0.14	0.16
N (countries)	115	115	115	115
Adjusted model	-4.78**	-7.34***	-0.66	1.72
	(-7.66,-1.90)	(-11.48,-3.19)	(-4.56,3.24)	(-2.26,5.70)
R^2	0.7	0.71	0.67	0.67
N (countries)	115	115	115	115

[1] All values are βs (95% CIs) derived via least squares regressions. RCP is the relative caloric price, as defined in the main text. Unadjusted models specify no controls, while the adjusted model controls for GDP per capita, the urban population share, the 15+years female labor force participation rate, and the 15+years female literacy rate, all specified in logs.
*,**,*** Significant differences: ***P<0.001, **P<0.01,*P<0.05.

Supplementary data

Supplemental Table S7. Cross-country robust regression estimates of the associations between stunting prevalence (HAZ<-2) among children 0-59 months and animal-sourced foods in models that adjust for RCPs for multiple food groups[1]

	Model 1	Model 2	Model 3	Model 4	Model 5
Milk prices, log	8.53*	7.79**	7.20*	5.08*	6.66***
	(1.88,15.18)	(2.21,13.38)	(1.62,12.79)	(0.18,9.97)	(2.91,10.41)
Egg prices, log	-2.49	-1.71	-3.05		
	(-9.26,4.28)	(-7.70,4.28)	(-8.81,2.71)		
Fish/meat prices, log	-4.15	-3.64			-4.11
	(-10.17,1.86)	(-9.01,1.74)			(-9.16,0.94)
Infant cereal prices, log	0.69			-0.64	
	(-4.11,5.49)			(-5.05,3.78)	
R^2	0.73	0.73	0.72	0.72	0.73
N	85	88	88	85	88

[1] All values are βs (95% CIs) derived via the robust regression command in STATA 14 (*rreg*) which downweighs outlying observations, although results are qualitatively similar with the least squares estimator. RCP is the relative caloric price, as defined in the main text. All models are adjusted for GDP per capita, the urban population share, the 15+years female labor force participation rate, and the 15+years female literacy rate, and the share of the population not using toilets (open defecation), all logged, but the models adjust for the RCPs of different food groups.
*,**,*** Significant differences: ***P<0.001, **P<0.01,*P<0.05.

Supplementary data

Supplemental Table S8. Cross-country robust regression estimates of the associations between severe, moderate and mild stunting among children 0-5 years and the logged Relative Caloric Prices (RCPs) of various animal-sourced foods in adjusted models[1]

	N=92<br>Milk RCPs, logged	N=92<br>Egg RCPs, logged	N=92<br>Meat/Fish RCPs, logged	N=89<br>Infant cereal RCPs, logged
		<u>Severe stunting (HAZ<-3)</u>		
	2.24**	1.71	0.91	1.67*
	(0.60,3.89)	(-0.07,3.49)	(-1.41,3.23)	(0.04,3.29)
R^2	0.67	0.65	0.64	0.66
N (countries)	91	91	91	88
		<u>Moderate stunting (HAZ<-2)</u>		
	3.81**	2.84	1.22	2.88*
	(1.03,6.58)	(-0.13,5.80)	(-2.72,5.17)	(0.16,5.60)
R^2	0.74	0.72	0.71	0.73
N (countries)	91	91	91	88
		<u>Mild stunting (HAZ<-1)</u>		
	3.80*	1.75	-0.72	2.04
	(0.09,7.51)	(-2.24,5.74)	(-5.76,4.33)	(-1.64,5.72)
R^2	0.7	0.67	0.68	0.68
N (countries)	91	91	91	88

[1] All values are βs (95% CIs) derived via the robust regression command in STATA 14 (*rreg*) which downweighs outlying observations, although results are qualitatively similar with the least squares estimator. RCP is the relative caloric price, as defined in the main text. Unadjusted models specify no controls, while the adjusted model controls for GDP per capita, the urban population share, the 15+years female labor force participation rate, and the 15+years female literacy rate, and the share of the population not using toilets (open defecation), all logged.

*,**,*** Significant differences: ****P*<0.001, ***P*<0.01,**P*<0.05.

Supplementary data

Supplemental Table S9. Cross-country least squares regression estimates of the associations between stunting prevalence among children 0-5 years and the relative calories costs of animal-sourced foods and fortified infant cereals in unadjusted and adjusted models[1]

	Milk RCP, logged	Egg RCP, logged	Meat/fish RCP, logged	Infant cereal RCP, logged
Unadjusted model	12.09***	11.15***	8.36**	10.52***
	(9.29,14.89)	(7.86,14.44)	(2.62,14.10)	(7.54,13.51)
R^2	0.43	0.32	0.08	0.35
N (countries)	100	100	100	94
Adjusted model	4.13**	2.48[#]	0.12	2.13
	(1.37,6.89)	(-0.39,5.35)	(-3.72,3.95)	(-0.68,4.94)
R^2	0.74	0.73	0.72	0.72
N (countries)	100	100	100	94

[1] All values are βs (95% CIs) derived via least squares regressions. RCP is the relative caloric price, as defined in the main text. Unadjusted models specify no controls, while the adjusted model controls for GDP per capita, the urban population share, the 15+years female labor force participation rate, and the 15+years female literacy rate, and the share of the population not using toilets (open defecation), all logged. [#],*,**,*** Significant differences: ***P<0.001, **P<0.01,*P<0.05, [#]P<0.10.

Supplementary data

Supplemental Table S10. Cross-country robust regression estimates of the associations between moderate stunting prevalence (HAZ<-2) among children 0-5 years and the prices of various vegetal foods in unadjusted and adjusted models[1]

	Other fruit RCP, logged	vitamin-A rich fruit & vegetables RCP, logged	Dark green leafy vegetables RCP, logged	Legume/nut RCP, logged
Unadjusted model	5.88*	6.64*	-5.92**	0.31
	(1.32,10.44)	(1.13,12.14)	(-10.29,-1.55)	(-5.07,5.68)
R^2	0.06	0.06	0.05	0.01
N (countries)	90	90	90	90
Adjusted model	-0.8	2.15	-3.13*	0.12
	(-3.49,1.90)	(-1.08,5.38)	(-5.68,-0.59)	(-2.79,3.03)
R^2	0.70	0.70	0.70	0.70
N (countries)	90	90	90	90

[1] All values are βs (95% CIs) derived via the robust regression command in STATA 14 (*rreg*) which downweighs outlying observations, although results are qualitatively similar with the least squares estimator. RCP is the relative caloric price, as defined in the main text. Unadjusted models specify no controls, while the adjusted model controls for GDP per capita, the urban population share, the 15+years female labor force participation rate, and the 15+years female literacy rate, and the share of the population not using toilets (open defecation), all logged.

*,**,*** Significant differences: ***P<0.001, **P<0.01,*P<0.05.

Supplementary data
